# AQB improves carboplatin sensitivity in endometrial cancer through dual DNA repair modulation: suppression of the p21-E2F1-RAD51 and ATF3-HDAC1-BRCA1 signaling

**DOI:** 10.1038/s41419-025-08287-4

**Published:** 2025-12-06

**Authors:** Xingyu Zheng, Eryan Yang, Ye Yan, Shuangshuang Zhao, Xianxian Li, Tianqi Wang, Yuening Piao, Wei Liu, Jiaxin Chen, Sitong Wen, Chao Gao, Wenyan Tian, Fengxia Xue, Chunsheng Kang, Yingmei Wang

**Affiliations:** 1 Department of Gynecology and Obstetrics, Tianjin Medical University General Hospital, Tianjin, China; 2 Tianjin Key Laboratory of Female Reproductive Health and Eugenics, Tianjin Medical University General Hospital, Tianjin, China; 3 Laboratory of Neuro-oncology, Tianjin Neurological Institute, Department of Neurosurgery, Tianjin Medical University General Hospital, Key Laboratory of Post-Neuro Injury Neuro-Repair and Regeneration in Central Nervous System, Ministry of Education and Tianjin City, Tianjin, China

**Keywords:** Endometrial cancer, Chemotherapy

## Abstract

Endometrial cancer (EC) is an increasingly common malignancy among women, and associated mortality rates continue to rise. Preferred treatment options for advanced or recurrent EC patients include a combination of carboplatin and paclitaxel, with modest clinical outcomes. Chemoresistance and drug toxicity are important factors that significantly affect the clinical efficacy of carboplatin. Therefore, there is an urgent need for therapeutic strategies that enhance carboplatin sensitivity, reduce its dose while maintaining efficacy, and ensure treatment safety. This study identified the novel small-molecule inhibitor AC1Q3QWB (AQB) as a potent enhancer of carboplatin efficacy. AQB disrupts the binding of HOTAIR to EZH2 and upregulates a series of tumor suppressor genes, such as *CDKN1A*, *ATF3*, and *BBC3*, thereby epigenetically suppressing the homologous recombination repair (HRR) pathway in EC, causing cell cycle arrest and inducing apoptosis. AQB inhibits carboplatin-induced RAD51 expression via the p21-E2F1 axis. Additionally, AQB epigenetically silences BRCA1 via ATF3-HDAC1 interactions at the BRCA1 promoter. In vivo studies using subcutaneous xenografts and a stage IV EC patient-derived xenograft (PDX) model demonstrated that AQB enhanced carboplatin’s antitumor effects, reduced the required carboplatin dose, and alleviated associated toxicity. The combination of AQB with standard chemotherapy holds promise for improving outcomes in patients with advanced or recurrent EC.

**Figure:**
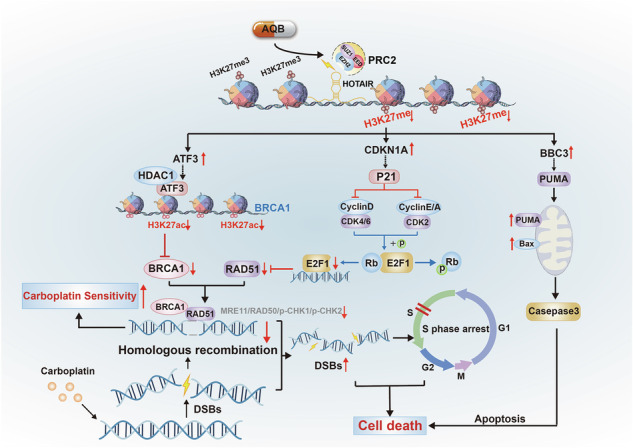
The schematic diagram illustrates the mechanism by which AQB enhances the sensitivity of EC cells to CBPt.

The schematic diagram illustrates the mechanism by which AQB enhances the sensitivity of EC cells to CBPt.

## Introduction

Endometrial cancer (EC) is the most common malignancy of the female reproductive system. Over the past three decades, its incidence has increased by 132%, with mortality rates rising annually and a trend towards younger patients, posing a serious threat to women’s health [1]. Although surgical resection and platinum-based chemotherapy provide effective disease control for the majority of patients, the prognosis for those with advanced, recurrent, or rare subtypes remains poor, with 5-year survival rates of 10–19% [2, 3]. Current first-line therapy for advanced or recurrent EC involves a combination of carboplatin (CBPt) and paclitaxel; however, the limited efficacy of this regimen results in a median survival of less than 3 years for treated patients [4]. Although patients often demonstrate initial sensitivity to platinum-based chemotherapy, most patients require additional lines of chemotherapy following disease progression [5]. The sensitivity to chemotherapy drugs and the reduction of their toxicity remain major challenges in the treatment of advanced or recurrent EC [6, 7]. Therefore, the development of more effective strategies to enhance the sensitivity of EC tumor cells to platinum-based chemotherapy remains an urgent priority.

CBPt exerts its effects by covalently binding to DNA, resulting in DNA crosslinking and adduct formation. This process induces DNA damage, disrupts normal transcription and replication, and ultimately triggers apoptosis. The sensitivity of tumor cells to platinum-based chemotherapeutic agents is predominantly determined by their capacity to detect and repair damage [8]. Double-strand breaks (DSBs) are the most deleterious form of damage to the DNA, and DSB repair is primarily mediated by the homologous recombination repair (HRR) pathway following platinum-based chemotherapeutic drug exposure. This pathway enables the repair of DSBs following the excision of platinum adducts without introducing any errors [9]. BRCA1 recruitment to DSBs occurs after their formation, whereupon it interacts with and enhances the activity of RAD51, the core recombinase, bringing it to the DSB site and controlling DNA end resection activity to crucially shape DSB repair [10]. Tumor cells deficient in BRCA1 or RAD51, or those with compromised DNA repair mechanisms, cannot effectively repair DNA damage and thus show heightened sensitivity to platinum-based chemotherapeutic agents. High RAD51/BRCA1 activity is linked to platinum tolerance, and RAD51 accumulation is widely used as a functional biomarker of HR proficiency [11]. Inhibition of HRR after DNA damage induced by platinum-based treatments is therefore crucial for maximizing the therapeutic efficacy of these agents.

Long noncoding RNAs (lncRNAs) are important regulators of diverse processes in human cells, including cell cycle progression, drug resistance, epigenetic control, the remodeling of the chromatin, and tumorigenesis [12]. The lncRNA HOTAIR has been reported to function as a molecular scaffold, binding to and facilitating the interaction between the polycomb repressive complex 2 (PRC2) and chromatin. This interaction suppresses transcriptional activity at target gene loci by promoting the trimethylation of histone H3 at lysine 27 (H3K27me3). This leads to global alterations in gene expression levels and epigenetic repression of tumor suppressor genes [13]. The core PRC2 component Enhancer of Zeste Homolog 2 (EZH2) is vital for H3K27 trimethylation [14]. HOTAIR and EZH2 have been implicated in significant pro-tumorigenic roles across various cancer types [15, 16], and their expression is closely associated with sensitivity to platinum-based chemotherapy [17, 18]. Elevated levels of HOTAIR and EZH2 are commonly observed in EC tissues, and their overexpression is often correlated with poorer patient outcomes [19, 20]. A few studies in EC have provided preliminary evidence that EZH2 silencing or inhibition can enhance the sensitivity of EC cells to cisplatin [20], and EZH2 inhibition has also been reported to improve cisplatin response in chemotherapy-resistant, ARID1A loss-of-function patient-derived xenograft models [21].Nevertheless, investigations specifically targeting HOTAIR or EZH2 to improve chemotherapy response in EC remain scarce, highlighting an important area for further exploration. In a previous study, our group determined that AC1Q3QWB (AQB), a small molecule inhibitor that targets the interaction between HOTAIR and EZH2, restores the expression of tumor suppressor genes such as *APC2*, *SOX17*, *P21*, *TP73*, *SFN*, *BBC3*, and *GADD45G* in EC, thereby inhibiting EC progressio [22]. The selective disruption of HOTAIR-EZH2 interactions is thus a promising approach to modulating the epigenomic regulation in EC, thereby optimizing combination treatment strategies with platinum-based drugs.

In this study, the significant upregulation of *ATF3*, *CDKN1A*, and *BBC3* were observed upon AQB treatment in EC cells as a consequence of the blockade of the HOTAIR-EZH2 interaction. AQB suppressed CBPt-induced RAD51 upregulation through the p21-E2F1 pathway. AQB also epigenetically silenced BRCA1 via ATF3-HDAC1 interactions at the BRCA1 promoter. AQB effectively suppressed HRR activity induced by CBPt, leading to more extensive CBPt-induced DNA damage, S-phase arrest, and tumor cell apoptosis. In vivo, AQB enhanced the antitumor efficacy of CBPt.

## Materials and methods

### Clinical data and patient samples

This study collected data from 127 EC patients who underwent platinum-based chemotherapy at the Department of Obstetrics and Gynecology, Tianjin Medical University General Hospital, between 2019 and June 2024. Myelosuppression was evaluated in these patients. All EC specimens were obtained from patients who were surgically treated at the same hospital (94 patients in Stage I, 7 patients in Stage II, 21 patients in Stage III, and 5 patients in Stage IV). The study was approved by the Institutional Review Board of Tianjin Medical University General Hospital, and written informed consent was obtained from all participants.

### Cell culture, drugs, and lentiviruses

Cell lines (HEC-1A and HEC-1B human EC cells) were provided by the Cell Resource Center at Peking Union Medical College. HEC-1A cells were cultured in McCoy’s 5 A Medium (Gibco, USA) containing 10% fetal bovine serum (FBS). HEC-1B cells were cultured and maintained in MEM (Gibco) with 10% FBS. All cells were cultured in a 5% CO_2_ incubator at 37 °C. AQB was procured from WuXi AppTec (Shanghai, China), while CBPt (HY-17393) was obtained from MedChemExpress (USA). AQB was dissolved in dimethyl sulfoxide (DMSO), and CBPt was resuspended in sterile deionized water (ddH_2_O). Knockdown of CDKN1A, ATF3, E2F1, and BBC3 was achieved using shRNA-containing lentiviral plasmids (sh-CDKN1A, sh-ATF3, sh-E2F1, sh-BBC3; GENECHEM, Shanghai, China), and HDAC1 was silenced by small interfering RNA (si-HDAC1; GENCEFE, China). All sequences are listed in Supplementary Table S1. Stable transfectants were selected with 2 μg/mL puromycin, and siRNA transfections were performed using Lipofectamine 3000 (Thermo Fisher Scientific). For gene overexpression, E2F1 was introduced using a lentiviral plasmid (OE-E2F1; GENECHEM), and a doxycycline-inducible lentiviral plasmid (Lenti-Dox-ATF3; IBSBIO, Cat# GM-LC-127067) was employed to achieve controlled ATF3 overexpression.

### Viability, colony formation, and flow cytometry analyses

A Cell Counting Kit-8 (CCK-8) assay (Dojindo, Japan) was used to evaluate cell viability. Before drug treatment, cells (3 × 10^3^/well) were seeded into 96-well plates. After drug treatment for a specified duration, cells were incubated with the CCK-8 reagent for 2 h. Absorbance (OD) at 450 nm was measured using a microplate reader. Cells (500/well) were plated into 6-well plates for colony formation assays and treated for 14 days. Colonies were then fixed with 4% paraformaldehyde (PFA) and stained with crystal violet for 15 min. Cell cycle distribution and apoptosis induction were evaluated *via* flow cytometry. Staining was performed with the Cell Cycle and Apoptosis Assay Kit (Beyotime) and the FITC Annexin-V/7-AAD Kit (BD Pharmingen), followed by analysis with a flow cytometer.

### RNA sequencing

RNA sequencing and corresponding analyses were performed for 12 HEC-1b cell RNA samples by Novogene Co., Ltd. (China).

### Western blotting (WB)

WB was performed as reported previously [22], using the primary antibodies listed in Supplementary Table S2. Unless otherwise specified, cells were harvested after 48 h of drug treatment for protein extraction. To evaluate the time-dependent dynamics of DNA damage and repair markers, cells were also collected at 24, 48, and 72 h after treatment.

### RT-qPCR

Total RNA was extracted using TRIzol (Invitrogen, USA), and 2 μg of RNA per sample was used to synthesize cDNA with the RevertAid First Strand cDNA Synthesis Kit (Thermo Scientific) according to the manufacturer’s guidelines. Quantitative PCR (qPCR) was performed using SYBR Green qPCR Master Mix (Selleck) and a QuantStudio 3 Real-Time PCR System (Thermo Scientific). Unless otherwise specified, cells were harvested after 24 h of drug treatment for RNA extraction. To assess the time-dependent effects on gene expression, samples were additionally collected at 24, 48, and 72 h as indicated. Relative gene expression was calculated using the 2^-ΔΔCt^ method. The primer sequences used in this study are listed in Supplementary Table S1.

### Chromatin immunoprecipitation (ChIP)

The SimpleChIP® Plus Sonication Chromatin IP Kit (CST, Cat# 56383S) was used as directed for ChIP assays, using antibodies specific for H3K27me3, E2F1, ATF3, and HDAC1. After precipitation, qPCR was used to analyze the purified DNA using the primers listed in Supplementary Table S1.

### Immunofluorescence (IF) staining

For IF detection of γ-H2AX, RAD51, and cleaved caspase-3, cells were collected after 48 h of drug treatment. EC cells were fixed with 4% PFA for 20 min at room temperature, permeabilized with 0.5% Triton X-100, and blocked for 1 h with 5% bovine serum albumin. Cells were then incubated overnight at 4 °C with primary antibodies specific for γ-H2AX, RAD51, and cleaved caspase-3. F-actin was stained using Alexa Fluor™ 488 phalloidin (Invitrogen, Cat# A12379), and primary antibodies were detected with Goat anti-Rabbit IgG Alexa Fluor™ 594 (Invitrogen, Cat# A-11012). Samples were mounted with a DAPI-containing antifade medium (Solarbio, Cat# S2110), and images were captured using an Olympus FluoView 1200 confocal microscope (Olympus, Japan). The primary antibodies used in these experiments are listed in Supplementary Table S2.

### In vivo xenograft model

All animal experiments described in this study were approved by the Institutional Animal Care and Use Committee of Tianjin Medical University. A subcutaneous model of EC was developed by implanting EC cells (HEC-1B) subcutaneously into 4-week-old BALB/c nude mice. Treatment was initiated 7 days post-implantation, and the animals were randomly assigned to 5 groups for a 2-week treatment period: (1) a control group receiving intraperitoneal injections of 10% DMSO; (2) an AQB group treated with 50 mg/kg AQB every two days; (3) a CBPt group treated with 50 mg/kg CBPt weekly; (4) a combination group treated with 50 mg/kg CBPt weekly and 50 mg/kg AQB every two days; and (5) a low-dose CBPt combination group treated with 25 mg/kg CBPt weekly and 50 mg/kg AQB every two days. Tumor sizes were monitored regularly during the study, and tumors were excised and weighed after euthanasia. Tumor volume was calculated using the formula (length × width²)/2.

To further evaluate the functional relevance of target genes, subcutaneous xenograft models were generated by injecting HEC-1B cells stably transfected with sh-NC, sh-CDKN1A, sh-ATF3, or sh-BBC3 into 4-week-old BALB/c nude mice. Seven days after implantation, the mice were randomly assigned to the following groups: sh-NC, sh-CDKN1A, sh-ATF3, sh-BBC3, AQB+CBPt, sh-CDKN1A + AQB+CBPt, sh-ATF3 + AQB+CBPt, and sh-BBC3 + AQB+CBPt. Drug administration followed the same regimen as above, with 50 mg/kg AQB intraperitoneally every two days and 50 mg/kg CBPt intraperitoneally once weekly. Tumor growth was monitored, and tumors were harvested and weighed after euthanasia. Tumor volume was calculated using the same formula as above.

### Immunohistochemistry (IHC) and Hematoxylin and eosin (H&E) staining

After paraffin embedding, tissues were sectioned, deparaffinized, rehydrated, and processed for antigen retrieval and incubated for 20 min in citrate buffer at 100 °C. These tissues were then either stained with H&E or stained for IHC using an IHC Kit (ZSGB-BIO, Cat# PV-9000) based on the provided instructions. The primary antibodies used in this study are detailed in Supplementary Table S2.

### Patient-derived xenograft (PDX) modeling

All animal studies were approved by the Animal Care and Use Committee of Tianjin Medical University, and informed consent was obtained from all participants. To develop a PDX model, 6- to 8-week-old female C-NKG severe immunodeficient mice were subcutaneously implanted with fresh tumor tissue samples from EC patients treated at the Department of Obstetrics and Gynecology, Tianjin Medical University General Hospital. When the tumors reached an 800 mm³ volume, they were passaged by subcutaneously implanting tumor tissue samples (2 × 2 × 2 mm³) into the right forelimb of the mice. Once the tumors reached an average volume of 110 mm³, mice were randomly assigned to 3 groups using a stratified randomization method for a 21-day treatment regimen: (1) a control group treated with DMSO; (2) a CBPt group treated with 50 mg/kg CBPt weekly, and (3) a combination group treated with 25 mg/kg CBPt weekly and 50 mg/kg AQB every two days. Body weight and tumor size were regularly monitored throughout the study. After euthanasia, tumor tissues were collected and weighed.

### Statistical analyses

Data was statistically analyzed in GraphPad Prism 8.4.0. Results were derived from ≥3 replicate experiments and are presented as means ± SD. Functional analyses were performed *via* Student’s t-tests or ANOVA. The combination scores were calculated using SynergyFinder (Bliss Model) [20]. Gene Set Enrichment Analysis (GSEA) was conducted using the OmicStudio platform (https://www.omicstudio.cn/tool). TCGA data were examined using the Xiantao platform (https://www.xiantaozi.com), and pathway enrichment analyses were performed using CAMOIP (http://www.camoip.net). Statistical significance was defined as *P < 0.05, **P < 0.01, ***P < 0.001, and **P < 0.0001, with “ns” indicating no statistical significance.

## Results

### AQB and CBPt treatment synergistically inhibit EC cell viability in vitro

**Fig. 1:**
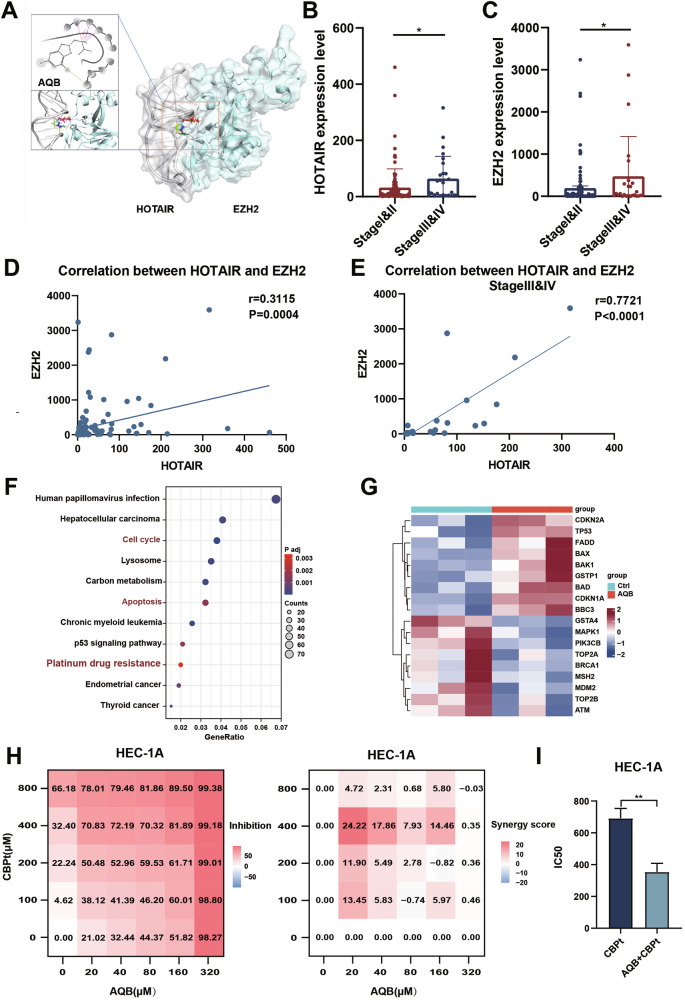
AQB and CBPt synergistically inhibit EC cell proliferation. **A** Molecular modeling of HOTAIR-EZH2 interaction and structural simulation of drug AQB. **B**, **C** Expression levels of HOTAIR and EZH2 in EC patients grouped by clinical stages (Stage I&II vs. Stage III&IV). **D** Correlation analysis between HOTAIR and EZH2 expression in EC patients, with (**E**) showing the correlation in advanced-stage EC patients (Stage III&IV). **F** KEGG analysis of DEGs between AQB and control groups. **G** Heatmap showing platinum drug resistance-related genes after AQB treatment. **H** Combination matrices of cell inhibition and synergy scores by AQB and CBPt. Data represent the mean of 3 independent experiments. **I** IC50 of CBPt after treatment with AQB. The data were expressed as the mean ± SD (n = 3). *P <0.05, **P < 0.01.

AQB was identified as a small molecule compound through high-throughput screening and molecular docking studies. It has been previously demonstrated to inhibit the progression of EC by disrupting the interaction between HOTAIR and EZH2, thus restoring the expression of multiple tumor suppressor genes. Furthermore, in vivo experiments conducted in nude mice confirmed the biological safety of AQB [22]. The structure of AQB is presented in Fig. 1A.

The high expression of HOTAIR and EZH2 has been associated with tumor progression and poor prognosis [23–25]. RT-qPCR analysis of RNA expression from 127 EC samples revealed that the levels of HOTAIR and EZH2 were elevated in advanced-stage (Stage III & IV) samples compared to early-stage (Stage I and II) samples (Fig. 1B and C). Furthermore, a positive correlation between the expression of HOTAIR and EZH2 was observed (*r* = 0.3115, P = 0.0004), which was significantly stronger in advanced-stage samples (r = 0.7721, P < 0.0001) (Fig. 1D and E). TCGA analysis also yielded similar results, showing elevated expression of HOTAIR and EZH2 in advanced EC (Stage III&IV) compared to early-stage cancer (Stage I&II), with a positive correlation between HOTAIR and EZH2 expression (r = 0.18, P = 0.019) (Supplementary Fig. S1A–C). These findings suggest that HOTAIR and EZH2 may play important roles in the progression of advanced EC, highlighting their potential as therapeutic targets.

The platinum-based chemotherapy regimen is the first-line treatment for the majority of patients with advanced or recurrent EC. In clinical practice, we assessed data from 127 EC patients who underwent platinum-based chemotherapy at our hospital between 2019 and June 2024. Myelosuppression results indicated that 71.2% of these patients showed some degree of myelosuppression, which was moderate-to-severe in 29.6% of patients. Bone marrow suppression incidence and severity rates elevated with increasing numbers of chemotherapy cycles. Long chemotherapeutic regimens (5–8 cycles) were associated with a higher rate of myelosuppression as compared to short regimens (1–4 cycles) (85.2% vs. 68.2%), and the same was true for the rate of moderate to severe myelosuppression (37.5% vs. 27.9%), emphasizing the cumulative toxic effects of this form of chemotherapeutic treatment (Supplementary Table S3). These observations underscore the need for strategies to mitigate chemotherapy-related toxicity.

Building on these findings and incorporating sequencing data that shows AQB modulates platinum drug resistance-related gene expression (Fig. 1F). Specifically, AQB was found to upregulate genes related to platinum resistance, such as *CDKN1A* and *BBC3*, while downregulating *BRCA1* (Fig. 1G). Therefore, we hypothesize that AQB, a small molecule drug targeting HOTAIR and EZH2, may enhance CBPt sensitivity, mitigate chemotherapy-related side effects, and ultimately improve treatment adherence in patients with advanced/recurrent EC, making it a promising candidate for combination therapy with platinum-based drugs. After assessing the IC50 values for AQB and CBPt across four commonly used EC cell lines, the CBPt-insensitive HEC-1A and HEC-1B cell lines were selected for further analyses (Supplementary Fig. S1D and E). In a dose-response matrix, CBPt and AQB showed synergistic inhibitory effects on the proliferation of both of these cell lines (Fig. 1H, Supplementary Fig. S1F). The treatment of EC cells with selected AQB concentrations decreased CBPt IC50 values to roughly half of the baseline (Fig. 1I, Supplementary Fig. S1G). These results suggested that AQB can enhance the sensitivity of EC cells to CBPt.

### AQB suppresses the HRR pathway to enhance CBPt sensitivity

**Fig. 2:**
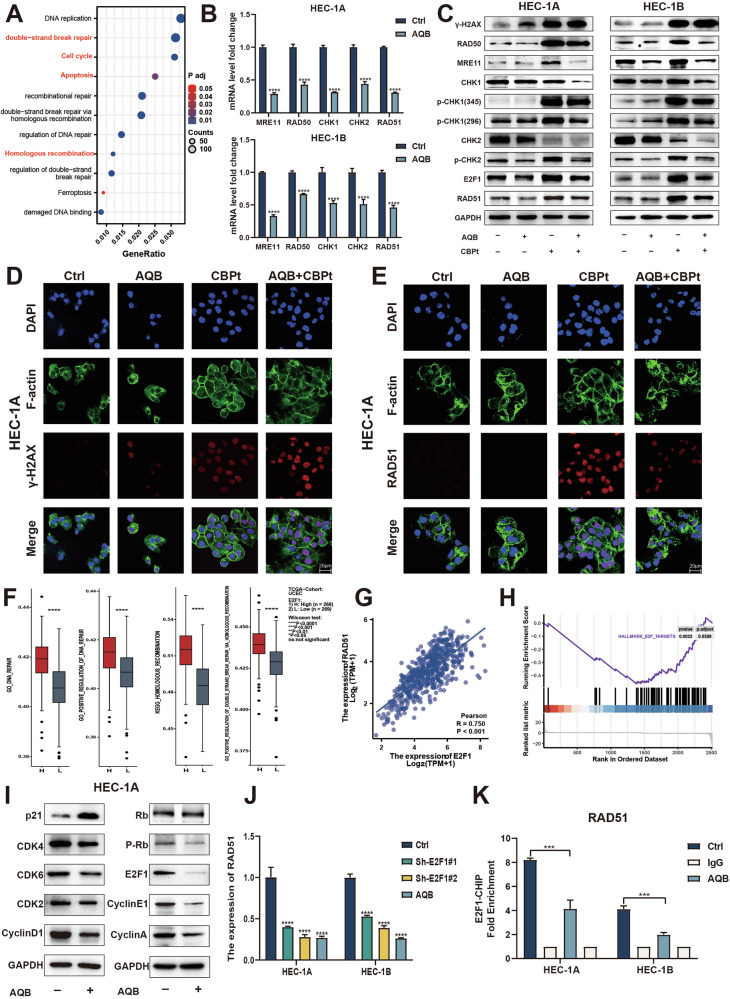
AQB inhibits the homologous recombination repair pathway. **A** KEGG and GO analysis of DEGs between AQB+CBPt and control groups. **B** mRNA levels of HR genes in cells treated with 80 μM AQB in HEC-1A and 60 μM AQB in HEC-1B for 48 h. **C** Western blotting of γ-H2AX and HR factors. **D, E** IF images of γ-H2AX and RAD51 in HEC-1A cells. Scale bar: 20 μm. **C**–**E**_ HEC-1A cells were treated with 200 μM CBPt, 80 μM AQB, or their combination for 48 h; HEC-1B cells were treated with 100 μM CBPt, 60 μM AQB, or their combination for 48 h. **F** Relationship between E2F1 expression and DNA repair and homologous recombination repair pathways in EC. **G** Scatter plot showing the correlation between E2F1 and RAD51 expression levels in EC. **H** GSEA analysis of DEGs between AQB and control groups. **I** Western blotting of p21 and its downstream proteins in HEC-1A cells. **J** mRNA levels of RAD51 in EC cells treated with AQB or shE2F1 for 48 h. **K** ChIP analysis of the RAD51 promoter region in EC cells using an anti-E2F1 antibody. The data are expressed as the mean ± SD (n = 3). ***P < 0.001, ****P < 0.0001.

The mechanisms by which AQB enhances CBPt sensitivity in EC were examined *via* RNA sequencing analysis of HEC-1B cells treated for 48 h with AQB, CBPt, or their combination (Supplementary Fig. S2A). Differentially expressed genes (DEGs) in the control and treatment groups are presented as volcano plots (Supplementary Fig. S2B–D). In GO and KEGG enrichment analyses of the DEGs identified when comparing the control and AQB + CBPt groups, significant enrichment of the “double-strand break repair,” “Cell cycle,” “Apoptosis,” and “Homologous recombination” pathways was observed (Fig. 2A). GO analyses of DEGs identified when comparing the control and AQB groups revealed significant enrichment in pathways, including “regulation of DNA repair” and “double-strand break repair *via* homologous recombination” (Supplementary Fig. S2E). Excessive activation of DNA damage repair (DDR) pathways reduces tumor sensitivity to chemotherapy drugs. DSBs, the most lethal form of DNA damage, are primarily repaired by the HRR pathway. These findings suggest that AQB may enhance EC cell sensitivity to CBPt by modulating HRR responses to this platinum-based drug.

To test this hypothesis, qPCR was used to detect the expression of canonical HRR-related genes (*RAD50, MRE11, CHK1, CHK2, RAD51*) [26]. After AQB treatment, a significant reduction in the mRNA levels of these genes was noted in EC cells (Fig. 2B). Upon treatment of these EC cell lines with AQB and/or CBPt, an increase in the DNA damage marker γ-H2AX was observed after CBPt treatment. However, a significant upregulation of HRR-related proteins, including RAD50, MRE11, p-CHK1 (ser345, ser296), p-CHK2, and RAD51, was detected in both cell lines after CBPt exposure. Strikingly, combined AQB and CBPt treatment increased the γ-H2AX levels in these cells while decreasing HRR-related protein levels compared to therapy with CBPt alone (Fig. 2C). To evaluate the persistence of AQB-mediated HRR inhibition, RAD51 and γ-H2AX expression were examined at 24, 48, and 72 h. AQB treatment persistently downregulated RAD51 at both the mRNA and protein levels (Supplementary Fig. S2F and G), while at all time points the AQB+CBPt combination produced significantly stronger γ-H2AX signals than CBPt alone (Fig. 2C, Supplementary Fig. S2H). These findings indicate that AQB persistently suppresses HRR and enhances CBPt-induced DNA damage, thereby increasing chemosensitivity. Confocal IF analyses further validated these findings (Fig. 2D and E, Supplementary Fig. S2I and J).

E2F1 is a transcription factor that plays a central role in coordinating DNA repair. Choi et al. determined that the depletion of E2F1 can reduce RAD51-mediated HRR and limit DNA damage-related cellular viability in colon cancer [27]. When the CAMOIP tool was used to conduct pathway enrichment analyses, E2F1 expression levels were significantly correlated with DNA repair efficiency in EC, with higher levels of E2F1 being linked to improved DNA DSB repair and HRR (Fig. 2F). Correlation scatter plots revealed a significant positive correlation between RAD51 and E2F1 expression in UCEC (Fig. 2G). WB analysis confirmed that E2F1 protein levels were elevated in CBPt-treated EC cells, suppressing this elevation by AQB (Fig. 2C). A GSEA analysis of DEGs identified when comparing the AQB-treated and control groups showed the downregulation of E2F protein target genes (Fig. 2H). We also previously reported the ability of AQB to upregulate the expression of CDKN1A through the inhibition of HOTAIR-PRC2-mediated H3K27me3 promoter enrichment [22]. In AQB-treated EC cells, these increases in p21 (encoded by *CDKN1A*) levels were closely related to the suppression of downstream factors involved in cell cycle regulation, such as the cyclinD1-CDK4/6 and cyclinA/E1-CDK2 complexes. These complexes are also important mediators of E2F1 and RB phosphorylation, prompting the assessment of the levels of total RB, phosphorylated RB (p-RB), and E2F1 upon AQB treatment. AQB-treated EC cells showed reduced p-RB and E2F1 levels (Fig. 2I, Supplementary Fig. S2K). This suggested that AQB suppressed the protein expression of E2F1 by upregulating p21. This led to the hypothesis that AQB may suppress HRR activity through the downregulation of E2F1 and consequent decreases in the expression of RAD51. Both the knockdown of E2F1 (Supplementary Fig. S2L and M) and treatment with AQB were sufficient to decrease RAD51 levels (Fig. 2J, Supplementary Fig. S2M). Overexpression of E2F1 in HEC-1A and HEC-1B cells was confirmed at the mRNA and protein levels (Supplementary Fig. S2N and O). Consistently, E2F1 overexpression significantly increased RAD51 expression, as shown by qPCR and WB analyses. Notably, AQB markedly reduced RAD51 expression in control cells, but this effect was largely abrogated under E2F1 overexpression, indicating that the suppressive effect of AQB on RAD51 is mediated through E2F1(Supplementary Fig. S2P and Q). In a ChIP assay, *RAD51* promoter E2F1 enrichment was reduced following AQB treatment (Fig. 2K). These results indicated that AQB induced p21 upregulation, in turn decreasing E2F1 expression, ultimately leading to the downregulation of E2F1-regulated RAD51 expression.

Given that AQB upregulates p21 and suppresses E2F1–RAD51 signaling, we investigated the role of the CDKN1A–E2F1 axis in AQB-mediated sensitization to CBPt. CDKN1A knockdown reduced CBPt sensitivity and weakened the inhibitory effects of AQB+CBPt on cell proliferation, while xenograft tumors derived from CDKN1A-knockdown cells grew faster and showed diminished responses to the combination treatment, consistent with Ki-67 IHC findings (Supplementary Fig. S3). In contrast, E2F1 knockdown enhanced CBPt sensitivity, whereas E2F1 overexpression reduced sensitivity (Supplementary Fig. S4A). Functionally, E2F1 overexpression attenuated the antiproliferative and pro-apoptotic effects of AQB+CBPt, restored RAD51 expression, and reduced γ-H2AX accumulation as well as the induction of Bax and cleaved caspase-3 (Supplementary Fig. S4B–E). Collectively, these results indicate that the CDKN1A–E2F1–RAD51 axis is a critical mediator of AQB-induced sensitization to CBPt in EC.

In summary, these results demonstrated that AQB can downregulate key HRR pathway-related factors induced by CBPt, including RAD50, MRE11, P-CHK1, P-CHK2, and RAD51. By downregulating E2F1, AQB is also capable of targeting the core HRR mediator RAD51, inhibiting HRR in response to CBPt and contributing to DNA damage accumulation, thereby rendering EC cells more sensitive to CBPt.

### AQB interacts with ATF3 and HDAC1 to achieve the epigenetic silencing of BRCA1

**Fig. 3:**
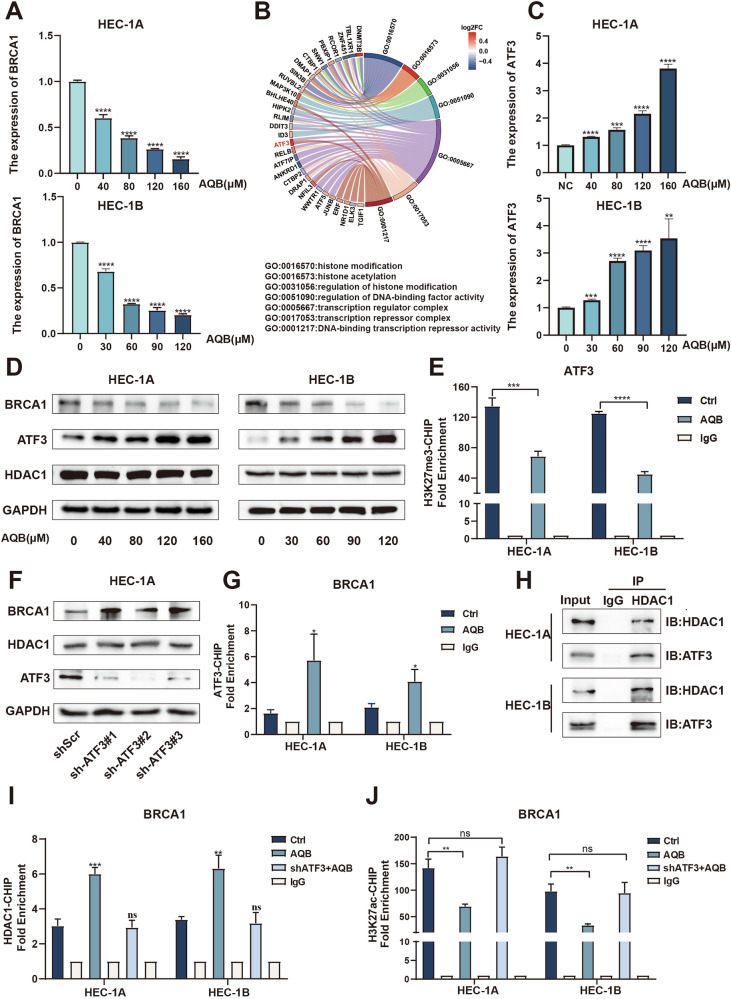
AQB inhibits BRCA1 expression by upregulating ATF3. **A** mRNA levels of BRCA1 in EC cells. **B** GO analysis of DEGs between AQB and control groups. **C** mRNA levels of ATF3 in EC cells. **D** Western blot analysis of ATF3, HDAC1 and BRCA1. **A**, **C**, **D** HEC-1A cells were treated with various concentrations of AQB (0, 40, 80, 120, and 160 μM) for 48 h, while HEC-1B cells were treated with AQB (0, 30, 60, 90, and 120 μM) for 48 h. **E** ChIP analysis of the ATF3 promoter region in EC cells using an anti-H3K27me3 antibody. **F** Protein expression levels of BRCA1 and HDAC1 after ATF3 knockdown in the HEC-1A cells. **G** ChIP analysis of the BRCA1 promoter region in EC cells using an anti-ATF3 antibody. **H** Co-IP assays were performed with anti-ATF3 antibody, followed by immunoblotting with HDAC1 antibodies. **I**, **J** ChIP analysis of the BRCA1 promoter region in EC cells using anti-HDAC1 and anti-H3K27ac antibodies. The data are expressed as mean ± SD (n = 3). *P < 0.05, **P < 0.01, ***P < 0.001, ****P < 0.0001, ns not significant.

BRCA1 plays a crucial role in sensitization to platinum-based chemotherapy through the repair of DSBs. When its function or expression is disrupted, this renders tumor cells incapable of reliably repairing the DNA damage induced by these chemotherapeutic drugs, culminating in apoptotic death [28, 29]. RNA sequencing revealed that AQB reduced BRCA1 expression within EC cells (Supplementary Fig. S5A). A dose-dependent drop in BRCA1 mRNA and protein levels was observed in AQB-treated HEC-1A and HEC-1B cells (Fig. 3A). In addition, both BRCA1 mRNA and protein levels progressively decreased at 24, 48, and 72 h following AQB treatment in these cells, indicating a sustained suppression of BRCA1 (Supplementary Fig. S5B and C). To examine the mechanism underlying AQB-induced BRCA1 downregulation, DEGs after treatment with this drug were analyzed. The results revealed significant enrichment in pathways related to “DNA-binding transcription repressor activity,” “transcription coregulator activity,” and “transcription corepressor activity,” with ATF3 being the most significant DEGs (Fig. 3B). ATF3 is a well-known transcriptional repressor that may stabilize inhibitory cofactor interactions with promoter regions, thereby suppressing the transcription of downstream genes [30]. This finding is noteworthy because the regulation of BRCA1 expression by ATF3 has not been reported, suggesting a potentially novel mechanism through which AQB modulates BRCA1 levels in EC cells.

Treatment with AQB dose-dependently induced ATF3 upregulation in EC cells at the mRNA level (Fig. 3C). Consistently, ATF3 protein levels increased in a dose-dependent manner in response to AQB, while BRCA1 levels showed the opposite trend in both HEC-1A and HEC-1B cells (Fig. 3D). Additionally, we speculated that the activation of ATF3 might result from AQB-mediated disruption of the HOTAIR-EZH2 interaction and a subsequent reduction in H3K27me3 accumulation at the promoter region of the gene. ChIP analysis confirmed a significant reduction in H3K27me3 enrichment at the *ATF3* promoter in these AQB-treated EC cells (Fig. 3E).

To explore the potential ability of ATF3 to regulate the expression of BRCA1 and the role that AQB plays in this process, ATF3 was knocked down in both EC cell lines, resulting in BRCA1 upregulation at the mRNA and protein levels (Supplementary Fig. S5D–F, Fig. 3F). Conversely, ATF3 overexpression in HEC-1A and HEC-1B cells led to a significant reduction of BRCA1 expression, as confirmed by qPCR and WB (Supplementary Fig. S5G–I). To further investigate this regulatory mechanism, ChIP assays also confirmed that, following AQB treatment, an increase in ATF3 enrichment was evident at the *BRCA1* promoter in EC cells (Fig. 3G). Studies have shown that HDAC1 inhibited the transcription of BRCA1 by modulating histone acetylation levels [31, 32]. A protein-protein interaction map indicated a connection between ATF3 and HDAC1 (Supplementary Fig. S5J). In Co-IP experiments, ATF3 was further confirmed to bind with HDAC1 (Fig. 3H), while in ChIP assays, an increase in HDAC1 enrichment was observed at the *BRCA1* promoter following AQB treatment. When ATF3 was knocked down in AQB-treated cells, no change in HDAC1 enrichment was observed (Fig. 3I). Moreover, HDAC1 expression remained unchanged with varying AQB treatment concentrations, ATF3 knockdown, or ATF3 overexpression in EC cells (Fig. 3D, F, Supplementary Fig. S5I). This supports a model in which ATF3 recruits HDAC1 to the *BRCA1* promoter after AQB treatment, forming a transcriptional repression complex that suppresses the *BRCA1* expression. Changes in *BRCA1* promoter H3K27ac levels were therefore assessed through a ChIP approach. Our results showed a decrease in H3K27ac occupancy after AQB treatment. However, when ATF3 was knocked down in EC cells treated with AQB, no change in H3K27ac levels was observed (Fig. 3J). To further validate the functional requirement of HDAC1 in this process, we silenced HDAC1 using three independent siRNAs, which efficiently reduced HDAC1 expression in both HEC-1A and HEC-1B cells (Supplementary Fig. S5K and L). As shown in Supplementary Fig. 5M and N, AQB treatment markedly reduced BRCA1 expression in both HEC-1A and HEC-1B cells. In contrast, knockdown of ATF3 or HDAC1 abolished this suppressive effect and resulted in a significant upregulation of BRCA1 expression. Moreover, combined knockdown of ATF3 and HDAC1 induced an even greater increase in BRCA1, indicating that AQB-mediated BRCA1 repression depends on the ATF3–HDAC1 axis.

Given that AQB represses BRCA1 via the ATF3–HDAC1 axis, we investigated whether ATF3 modulates AQB-mediated sensitization to CBPt. In dose–response assays, ATF3 knockdown decreased CBPt sensitivity, whereas ATF3 overexpression enhanced sensitivity (Supplementary Fig. S6A). Functionally, ATF3 knockdown attenuated the inhibitory effects of AQB+CBPt on proliferation, reduced apoptosis and γ-H2AX accumulation, and diminished induction of Bax and cleaved caspase-3 (Supplementary Fig. S6B–F). In xenograft models, ATF3 knockdown compromised the antitumor efficacy of AQB+CBPt, as evidenced by accelerated tumor growth and higher Ki-67 staining (Supplementary Fig. S6G–K). Collectively, these findings indicate that ATF3 is required for AQB-mediated sensitization to CBPt.

Together, these findings confirm that HDAC1, together with ATF3, is indispensable for AQB-induced repression of BRCA1, and further demonstrate that AQB upregulates ATF3, which recruits HDAC1 to the BRCA1 promoter, leading to histone deacetylation, reduced H3K27ac levels, and transcriptional silencing of BRCA1.

### AQB enhances CBPt-induced S-phase arrest in EC cells

**Fig. 4:**
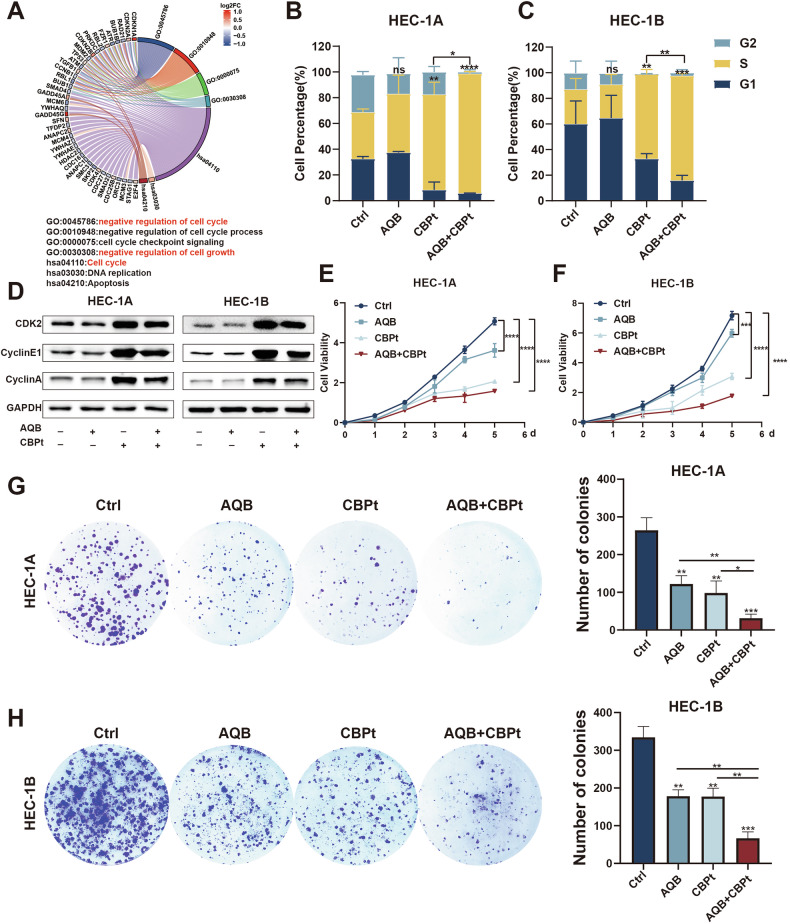
AQB Enhanced CBPt-Induced S Phase Arrest. **A** GO and KEGG analysis of DEGs between AQB and control groups. **B, C** Cell cycle distribution in EC cells. **D** Expression levels of S phase arrest-related proteins. **E, F** CCK8 assay in EC cells. **G, H** Colony formation assay in EC cells. **B**–**H** HEC-1A cells were treated with 80 μM of AQB,200 μM of CBPt, or 200 μM of CBPt +80 μM of AQB; HEC-1B cells were treated with 60 μM of AQB, 100 μM of CBPt, or 100 μM of CBPt +60 μM of AQB. Data are expressed as mean ± SD (n = 3). *P < 0.05, **P < 0.01, ***P < 0.001, ****P < 0.0001, ns not significant.

GAEA analyses revealed that AQB treatment negatively regulated the cell cycle and cell growth pathways (Fig. 4A). As DNA damage repair is closely tied to the cell cycle, a cell cycle analysis was next performed via flow cytometry. The treatment of EC cells with CBPt-induced S-phase cell cycle arrest was significantly enhanced by AQB (Fig. 4B and C). This observation suggests that AQB exacerbates DNA damage by inhibiting CBPt-induced HRR activity, resulting in irreparable damage and S-phase arrest. Cyclin synthesis and degradation are important for regulating the cell cycle. When the activities of Cyclin A/Cyclin E1 or CDK2 are inhibited, this leads to the stalling of DNA replication and consequent S-phase arrest [33–35]. WB analyses of proteins associated with S-phase arrest revealed a downregulation of CDK2 and Cyclin A/Cyclin E in the AQB+CBPt treatment group compared to the CBPt group (Fig. 4D). CCK8 and colony formation assays also revealed that combining AQB and CBPt led to the significant inhibition of EC cell proliferation, with this effect being more pronounced than that for either of these drugs individually (Fig. 4E–H). These findings indicate that AQB potentiates CBPt-induced S-phase arrest and that their combined application substantially enhances the inhibitory effect on cellular proliferation.

### Combination of AQB and CBPt enhances apoptosis in EC cells

**Fig. 5:**
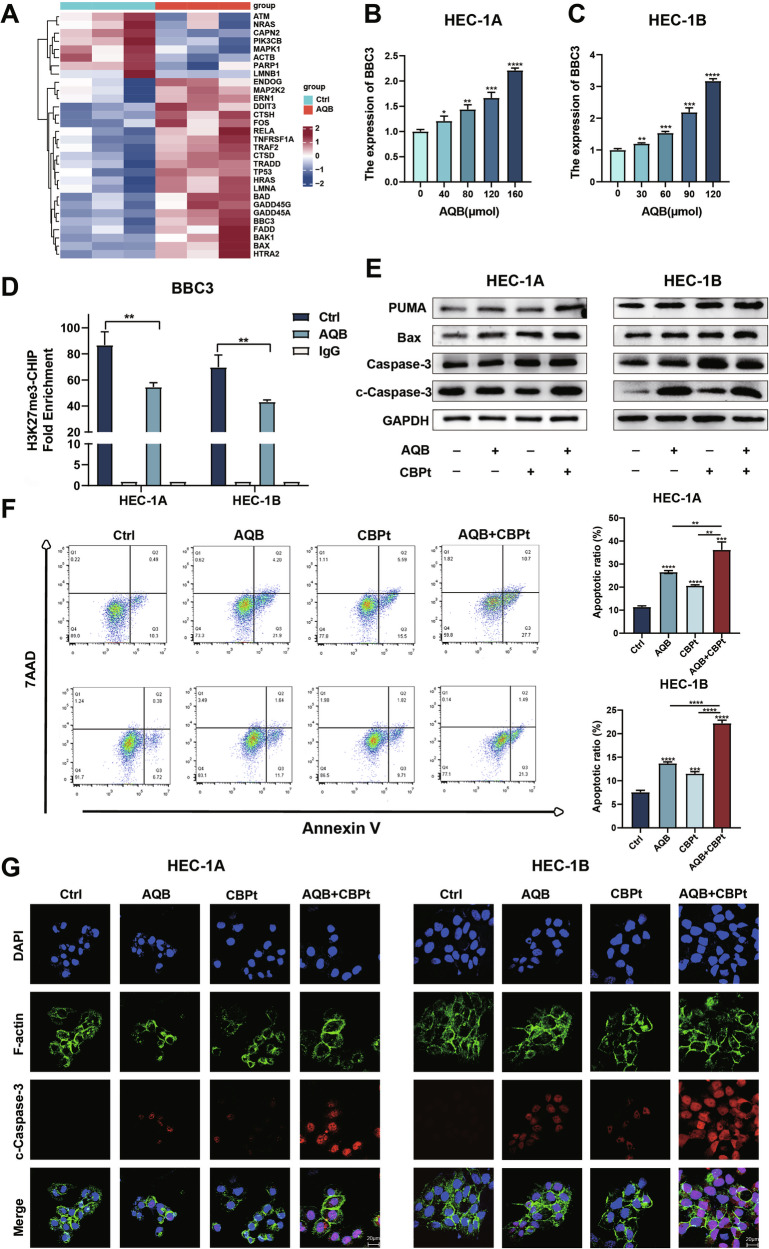
AQB and CBPt combination promotes apoptosis in EC cells. **A** Heatmap showing the apoptosis-related gene expression changes after AQB treatment. **B, C** HEC-1A cells were treated with various concentrations of AQB (0, 40, 80, 120, and 160 μM) for 48 h, while HEC-1B cells were treated with AQB (0, 30, 60, 90, and 120 μM) for 48 h. The mRNA levels of BBC3. **D** ChIP analysis of BBC3 promoter region in EC cells using an anti-H3K27me3 antibody. **E** Expression levels of apoptosis-related proteins. **F** The effect of AQB, CBPt, and AQB+CBPt on cell apoptosis analyzed by flow cytometry (**G**) IF images of cleaved-caspase3 in EC cells. Scale bar, 20 μm. **E**–**G** HEC-1A cells were treated with 200 μM of CBPt, 80 μM of AQB, or 200 μM of CBPt +80 μM of AQB for 48 h; HEC-1B cells were treated with 100 μM of CBPt, 60 μM of AQB, or 100 μM of CBPt +60 μM of AQB for 48 h. Data are expressed as mean ± SD (n = 3). *P < 0.05, **P < 0.01, ***P < 0.001, ****P < 0.0001.

RNA sequencing analyses identified that AQB regulates various apoptosis-related genes. Examination of genes involved in the positive regulation of apoptosis revealed upregulation of BBC3 (Fig. 5A). This finding was further validated by qPCR, which confirmed the dose-dependent upregulation of BBC3 in AQB-treated EC cells (Fig. 5B and C). To clarify the mechanisms underlying this finding, a ChIP assay was performed using an anti-H3K27me3 antibody, revealing decreased H3K27me3 enrichment of the BBC3 promoter in cells treated with AQB (Fig. 5D). WB analyses demonstrated significant increases in the levels of PUMA, a pro-apoptotic protein encoded by BBC3, in cells treated with both AQB and CBPt, together with the protein level upregulation of the downstream pro-apoptotic Bax and apoptotic effector cleaved caspase-3 (Fig. 5E). Flow cytometry indicated that both AQB and CBPt were individually capable of inducing EC cell apoptosis. This effect was significantly enhanced when both were used in combination (Fig. 5F). Confocal imaging demonstrated an increase in cleaved caspase-3 protein levels after combined AQB and CBPt treatment (Fig. 5G). These findings demonstrated the ability of AQB to enhance CBPt-induced apoptotic EC cell death through the upregulation of *BBC3*.

To further assess the role of BBC3 in AQB-mediated chemosensitization, stable knockdown of BBC3 was established in HEC-1A and HEC-1B cells, resulting in reduced PUMA expression (Supplementary Fig. S7A and B). BBC3 knockdown decreased CBPt sensitivity in dose–response assays (Supplementary Fig. S7C and D) and attenuated the inhibitory effects of AQB+CBPt on proliferation (Supplementary Fig. S7E and F). In xenograft models, BBC3 knockdown compromised the antitumor efficacy of AQB+CBPt, as evidenced by accelerated tumor growth and higher Ki-67 staining compared with controls (Supplementary Fig. S7G–K). Collectively, these findings demonstrate that BBC3 is required for AQB-mediated sensitization of EC cells and tumors to CBPt.

### AQB treatment enhances CBPt sensitivity while alleviating myelosuppression in vivo

**Fig. 6:**
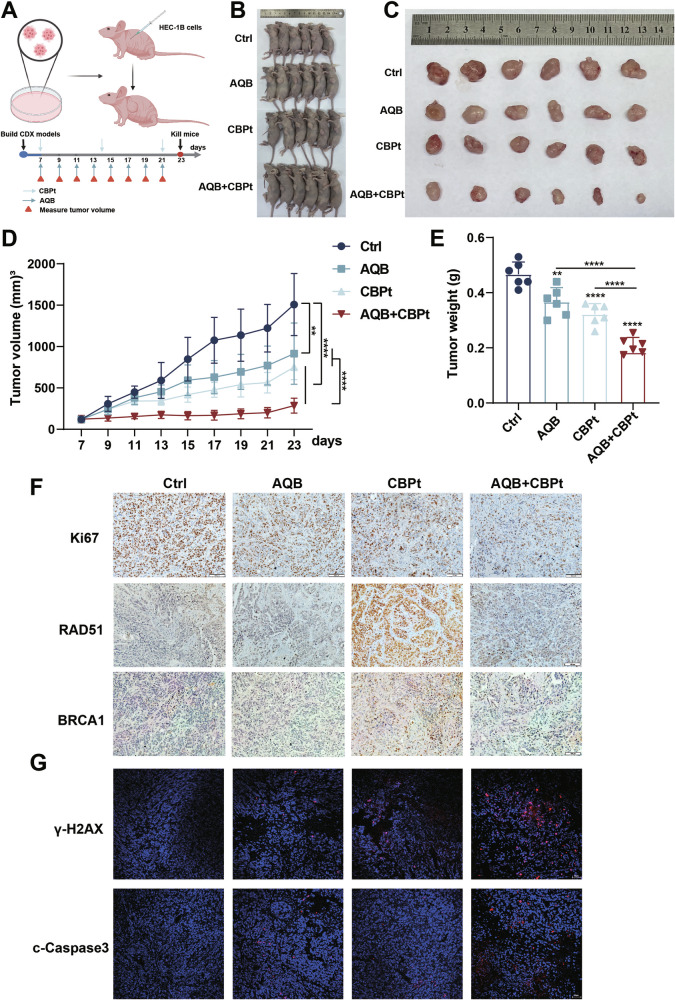
AQB enhances CBPt sensitivity in vivo. **A** Tumor-bearing nude mice were treated with DMSO, AQB (50 mg/kg), CBPt (50 mg/kg), or AQB (50 mg/kg) + CBPt (50 mg/kg) by intraperitoneal injection (Created with bioRender.com). **B** Image of mice after subcutaneous implantation of HEC-1B cells (n = 6 per group). **C** Excised tumors from each group. **D** Tumor volume changes over time(days). **E** Tumor weights at the end of treatment. **F** IHC staining for Ki67, RAD51 and BRCA1 in tumor tissues. Scale bars:100 μm. **G** IF staining of γ-H2AX and cleaved caspase3 in tumor tissues. Scale bars:50 μm. Data are expressed as mean ± SD (n = 6 mice). **P < 0.01, ****P < 0.0001.

To evaluate the potential translatability of these in vitro findings to an in vivo setting, HEC-1B cells were then subcutaneously implanted into nude mice to develop a xenograft tumor model. These mice were treated for 14 days with vehicle, AQB (50 mg/kg), CBPt (50 mg/kg), or a combination of AQB (50 mg/kg) and CBPt (50 mg/kg) (Fig. 6A). In these analyses, both AQB and CBPt effectively inhibited tumor growth; however, the reduction in tumor size was significantly more pronounced with the combined AQB + CBPt treatment than either treatment alone (Fig. 6B–E). IHC analyses demonstrated a reduction in Ki67 levels in the AQB+CBPt group relative to those in tumors from animals treated with CBPt alone. Tumor sections from CBPt-treated animals also presented with higher levels of HRR-related proteins, including RAD51 and BRCA1, whereas this increase was reduced in the AQB + CBPt group (Fig. 6F). Moreover, IF staining also highlighted a modest increase in nuclear γ-H2AX levels after CBPt treatment, whereas these levels were significantly higher after combination treatment. Combined therapy also resulted in higher levels of expression of the apoptotic effector protein cleaved-caspase-3 (Fig. 6G).

**Fig. 7:**
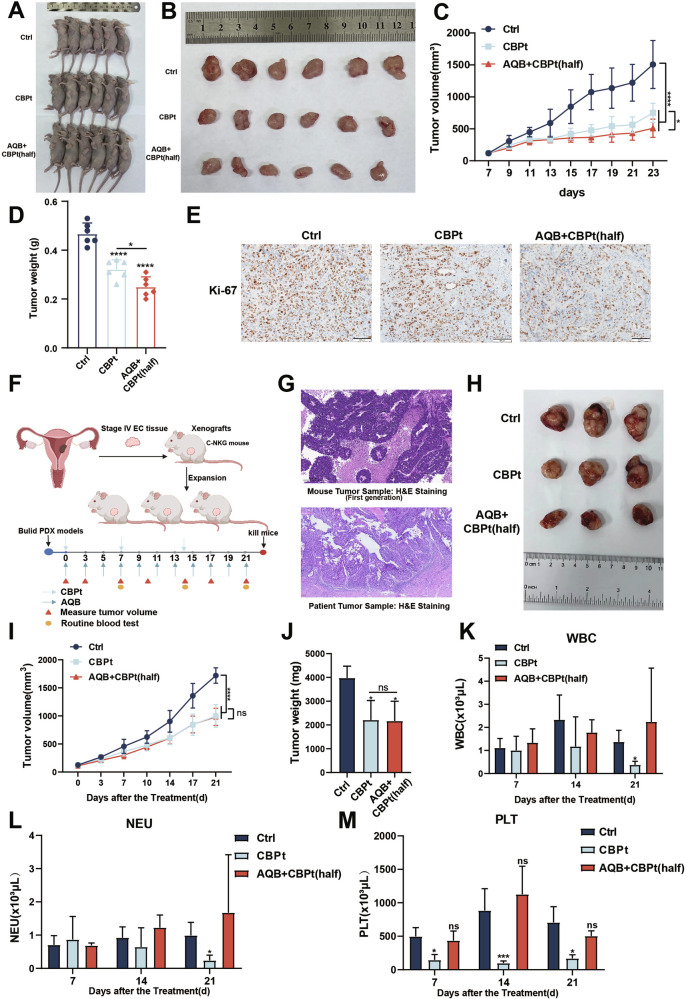
AQB enhances CBPt sensitivity and reduces myelosuppression. **A** Image of mice after subcutaneous implantation of HEC-1B cells (n = 6 per group). Tumor-bearing nude mice were treated with DMSO, CBPt(50 mg/kg) or AQB (50 mg/kg) + CBPt (25 mg/kg). **B** Excised tumors from each group. **C** Tumor volume changes over time. **D** Tumor weights at the end of treatment. **E** IHC staining for Ki67 in tumor tissues. Scale bars, 100 μm. **F** PDX models were established from stage IV EC patient tissues. Mice were randomly divided into 3 groups: DMSO, CBPt (50 mg/kg), or AQB (50 mg/kg) + CBPt (25 mg/kg) (Created with bioRender.com). **G** H&E-stained section of a PDX mouse tumor derived from an EC patient’s primary tumor sample and the original tumor sample from patient. **H** Excised tumors from each group. **I** Tumor volume changes over time. **J** Tumor weights at the end of treatment. **K** WBC, **L** NEU, and **M** PLT levels during treatment. Data are expressed as mean ± SD (n = 6 nude mice, n = 3 C-NKG mice) *P < 0.05, ***P < 0.001, ****P < 0.0001, ns not significant.

CBPt is a first-line treatment for various solid tumors; however, its therapeutic effect is frequently limited by myelosuppression and other dose-dependent toxicities [36]. Therefore, there is considerable potential in exploring dose optimization and combination treatment strategies that maintain therapeutic efficacy while minimizing associated side effects. In this context, the same batch of xenograft tumor-bearing mice were treated with a reduced-dose CBPt regimen consisting of AQB (50 mg/kg) + CBPt (25 mg/kg) to assess the potential of AQB in reducing CBPt dosage while preserving its antitumor efficacy (Fig. 7A). These results demonstrated that co-treatment with AQB and the reduced CBPt dose yielded therapeutic efficacy that was either comparable to or exceeded that achieved with the higher dose of CBPt alone (Fig. 7B–D). IHC analyses further indicated a significant reduction in Ki67 expression in the AQB + CBPt (half) treatment group, with this effect being comparable to that induced by the higher dose of CBPt alone (Fig. 7E).

To further extend these analyses, tumor tissue samples from stage IV EC patients treated in our hospital developed PDX models to simulate the actual growth conditions present within patient tumors (Fig. 7F). In this model system, the antitumor efficacy of combining AQB and CBPt was further investigated, along with the assessment of treatment-related side effects and safety. The development of the PDX model was confirmed through H&E staining of the first-generation parental tumors. These analyses demonstrated a high degree of similarity in the histological features of parental tumors as compared to those of the patient tumors from which they were derived (Supplementary Fig. S8A, Fig. 7G), confirming the ability of these models to simulate the patient tumor growth environment. Mice for this experiment were separated into a control group, a CBPt (50 mg/kg) group, and an AQB (50 mg/kg) + CBPt (25 mg/kg) group. In line with the earlier results, the antitumor activity of the combination AQB + CBPt (half) was similar to that of the higher dose used in the CBPt-only group (Supplementary Fig. S8B, Fig. 7H–J). Further, the body weight of mice in both the CBPt-alone group and the combination therapy showed insignificant changes compared to the control group (Supplementary Fig. S8C). Myelosuppression, characterized by decreases in white blood cells (WBC), neutrophils (NEU), and platelets (PLT), is a common side effect of CBPt treatment [37]. To evaluate this, routine blood tests were conducted on the respective mice on days 7, 14, and 21 after treatment. No significant differences in NEU or WBC counts were observed between the groups on days 7 or 14. However, on day 21, these levels were significantly lower in the CBPt group compared to the control group, whereas no such decrease was observed when comparing the AQB + CBPt (half) group to the control group (Fig. 7K and L). In the CBPt group, PLT levels were significantly reduced on days 7, 14, and 21, while no significant differences were observed between the AQB + CBPt (half) and control groups (Fig. 7M). No significant differences in other routine blood parameters were detected among the three groups (Supplementary Table S4). Histopathological examination through H&E staining revealed no significant target organ toxicity, and liver and kidney function tests showed no abnormalities, further confirming the absence of substantial organ toxicity (Supplementary Fig. S8D, Supplementary Table S5). These findings indicated that AQB is capable of reducing the required dose of CBPt while preserving antitumor efficacy, contributing to the robust mitigation of side effects including myelosuppression, thus translating to a better safety and tolerance profile. Collectively, these findings demonstrate that AQB enhances the sensitivity of EC tumors to CBPt in vivo.

## Discussion

EC is one of the most common gynecologic cancers. The global incidence and mortality of EC have consistently increased. Treatment outcomes remain poor in cases of advanced and recurrent EC [38]. While most patients have a favorable prognosis after early diagnosis and standard surgical treatment, approximately 25% of early-stage patients and more than 50% of advanced-stage patients experience recurrence [39]. Despite advances in cancer research emphasizing precise molecular profiling for EC diagnosis and treatment, which provide valuable insights into the development of novel treatment strategies [40, 41]. For all subtypes of advanced/recurrent EC, the 2025 National Comprehensive Cancer Network (NCCN) guidelines recommend first-line treatment consisting of CBPt combined with paclitaxel, along with immune checkpoint inhibitors [42]. Therefore, platinum-based chemotherapy, primarily CBPt, is still the cornerstone of therapy for advanced/recurrent EC. Chemoresistance, in particular, remains one of the primary barriers to platinum-based drug treatment [43], with CBPt being prone to developing resistance over time. Additionally, CBPt can cause significant myelosuppression, limiting its use in a full-dose, full-duration regimen [44, 45]. Therefore, enhancing CBPt sensitivity and reducing its side effects are crucial to improving treatment outcomes for advanced/recurrent EC. In this study, we found that the small molecule inhibitor AQB significantly enhances the sensitivity of EC to CBPt and effectively alleviates its side effects. This finding provides new insights into improving the treatment outcomes of advanced/recurrent EC, particularly in enhancing the efficacy of platinum-based drugs while reducing their systemic toxicity, holding potential for clinical application.

The primary mechanisms that shape sensitivity to platinum-based drugs include epigenetic changes, DDR induction, reduced active platinum species accumulation, and the impairment of the DNA damage-induced apoptosis pathway [8]. Epigenetic changes can provide insight into instability and non-genetic drug resistance. The H3K27 methyltransferase EZH2 is also involved in the acquisition of cisplatin and CBPt resistance by silencing tumor suppressor genes [46, 47]. Given its role in tumor chemoresistance, targeting epigenetic mechanisms with specific drugs offers a promising strategy to enhance the efficacy of platinum-based therapies. HOTAIR interacts with EZH2 to recruit the PRC2 complex, which catalyzes H3K27me3, thereby repressing the expression of tumor suppressor genes. This process restricts chromatin accessibility, silences the transcription of associated genes, and consequently promotes tumorigenesis and progression [23]. In our previous studies, we found that AQB, a small molecule drug targeting HOTAIR and EZH2, restores the expression of multiple tumor suppressor genes and inhibits EC progression, demonstrating significant antitumor activity and a favorable safety profile. Furthermore, we analyzed clinical samples from 127 EC patients in our hospital, along with TCGA data. The results showed that both HOTAIR and EZH2 are highly expressed in advanced EC and exhibit a significant positive correlation. This finding provides important clinical evidence supporting the critical roles of HOTAIR and EZH2 in EC progression and offers a theoretical basis for future research on advanced and recurrent EC. This study found that AQB regulates genes associated with platinum drug resistance. Notably, when combined with CBPt, AQB significantly inhibited the proliferation of EC cells. These findings present a novel strategy to enhance the effectiveness of platinum-based therapies in EC treatment and offer a promising combination therapy approach for clinical application.

The sensitivity of tumor cells to platinum-based chemotherapy is primarily determined by their ability to repair DNA damage induced by these drugs. With respect to DDR mechanisms, the impairment of HRR is a particularly important mechanism that can sensitize tumor cells to platinum-based therapy. By forming DNA adducts, these platinum drugs can induce DSB formation within cancer cells. The MRN complex (MRE11, RAD50, and NBS1) then recognizes these DSBs and activates the DDR pathway, triggering ATM, ATR, and CHK2/CHK1 activation together with a response mediated by BRCA1/2 and RAD51 that ultimately promotes the HRR of the damaged genomic material [48]. When DNA damage accumulates within cells without timely repair, this can trigger apoptotic death. If the damage is repaired sufficiently quickly, however, cells can survive, rendering them resistant to chemotherapeutic intervention. Overcoming this effect to induce tumor cell death is essential to potentiating the antitumor efficacy of platinum-based drugs. When apoptotic death is inhibited in tumor cells, dosing at traditional drug levels is insufficient to induce this form of cell death, similarly contributing to chemoresistance [49]. In our study, AQB was found to inhibit CBPt-induced expression of key proteins in the HRR cascade, including MRE11, RAD50, P-CHK1, P-CHK2, and RAD51, leading to DNA damage accumulation and promoting tumor cell death. Furthermore, AQB upregulated the expression of the BBC3 (PUMA) gene, further facilitating apoptosis.

To further investigate the role of AQB in regulating the HRR pathway, this study provides an in-depth analysis of its impact on key HRR-related molecules, particularly the regulation of RAD51 and BRCA1.RAD51 is a core protein of the HRR pathway, essential for the effective repair of double-strand breaks (DSBs). It primarily functions by promoting precise pairing and exchange between the damaged DNA strand and the intact homologous strand, enabling accurate DNA repair [50]. E2F1 has been demonstrated to facilitate DNA repair by promoting the recruitment of repair-related proteins to sites of DNA damage [51, 52]. Our results demonstrate that AQB upregulates CDKN1A (p21), downregulates E2F1, and suppresses RAD51, consistent with previous studies showing that E2F1 coordinates HR gene programs and directly transactivates RAD51 in response to DNA damage [53, 54]. Loss of E2F1 impairs RAD51-mediated HRR, highlighting its critical role in maintaining genomic stability [27]. Consistently, we found that AQB interferes with HOTAIR–EZH2 binding, thereby blocking CBPt-induced RAD51 upregulation and ultimately leading to HRR inhibition.BRCA1, another essential mediator of the HRR pathway, is responsible for DSB recognition, RAD51 loading, and ensuring repair accuracy [55]. Mutations or loss of BRCA1 can render tumor cells more sensitive to platinum-based chemotherapy, making it a key therapeutic target. ATF3 is a critical regulator of DNA damage response (DDR), stress-induced cellular processes, apoptotic pathways, and immune activity [56]. As a transcriptional repressor, ATF3 competes with other transcription factors for genomic binding or interacts with HDACs and co-repressors to inhibit transcription initiation, leading to target gene downregulation [30]. Our findings reveal that AQB upregulates ATF3 expression by disrupting the binding between HOTAIR and EZH2. Notably, ATF3 binds to the BRCA1 promoter and recruits HDAC1, resulting in the epigenetic repression of BRCA1. These findings highlight novel mechanisms by which AQB regulates key components of the HRR pathway in EC. By modulating RAD51 and BRCA1 activity, AQB disrupts this critical repair pathway, thereby enhancing their sensitivity to chemotherapy. Additionally, we identify a previously unreported role for ATF3 in the epigenetic regulation of BRCA1, providing new insights into modulating HRR and increasing chemotherapy sensitivity in EC.

By inhibiting the CBPt-induced HRR response, AQB triggered enhanced DNA damage accumulation in EC cells, resulting in S-phase growth arrest and apoptosis induction. We validated that AQB could enhance the antitumor effect of CBPt in EC xenograft models. Furthermore, we constructed a PDX model using clinical samples from advanced EC patients, providing a more accurate representation of late-stage disease. This model demonstrated that AQB reduces the required dose of CBPt while alleviating CBPt-associated myelosuppression. Given that myelosuppression is a frequent dose-limiting toxicity of carboplatin-based regimens in EC [57, 58] these findings highlight the promise of AQB as a potential adjuvant strategy to improve treatment tolerability and efficacy, providing a rationale for further preclinical evaluation and eventual clinical investigation.

AQB enhances platinum sensitivity in EC primarily by suppressing HRR through RAD51 downregulation and BRCA1 epigenetic repression. Based on this mechanism, it may also synergize with other HRR-targeting agents. In line with this, our preliminary data showed that AQB combined with Olaparib (PARP inhibitors) exerted synergistic effects on growth inhibition and apoptosis in EC cells. These results suggest that, beyond platinum-based chemotherapy, AQB holds potential for broader application in combination with HRR-targeted therapies, providing a promising direction for future investigation.

In summary, the results of this study revealed that AQB enhances the efficacy of CBPt and increases its sensitivity by targeting multiple pathways and key molecular targets, thereby inhibiting EC cell proliferation and survival. This study offers novel insights into the potential utility of combining epigenetic drugs and conventional chemotherapeutic agents in order to treat advanced/recurrent EC.

### Grant Support

The project was supported by the grants (Nos. 82172626 and 82202979) from the National Natural Science Foundation of China, by the grant (No. TJWJ2022XK009) from the Key Research Program of Tianjin Health Commission, by the grant from the Tianjin Natural Science Foundation for Youth (No. 23JCQNJC00530), by the Beijing Science and Technology Innovation Medical Development Foundation (No. KC2023-JX-0288-RZ139), and by the Tianjin Key Medical Discipline (Specialty) Construction Project (Program No. TJYXZDXK-031A).

## Supplementary information

supplementary material

Western blot data.

Supplementary Excel file 1

Supplementary Excel file 2

## Data Availability

The datasets used and/or analyzed during the current study are available from the corresponding author on reasonable request.
