# Supplementary material for: AQB improves carboplatin sensitivity in endometrial cancer through dual DNA repair modulation: suppression of the p21-E2F1-RAD51 and ATF3-HDAC1-BRCA1 signaling
Source: Cell Death Dis. 2025 Dec 6;17(1):70. doi: 10.1038/s41419-025-08287-4 (PMC12828040; doi:10.1038/s41419-025-08287-4)
Supplement: Supplementary file 1 — supplementary material [file 41419_2025_8287_MOESM1_ESM.pdf]

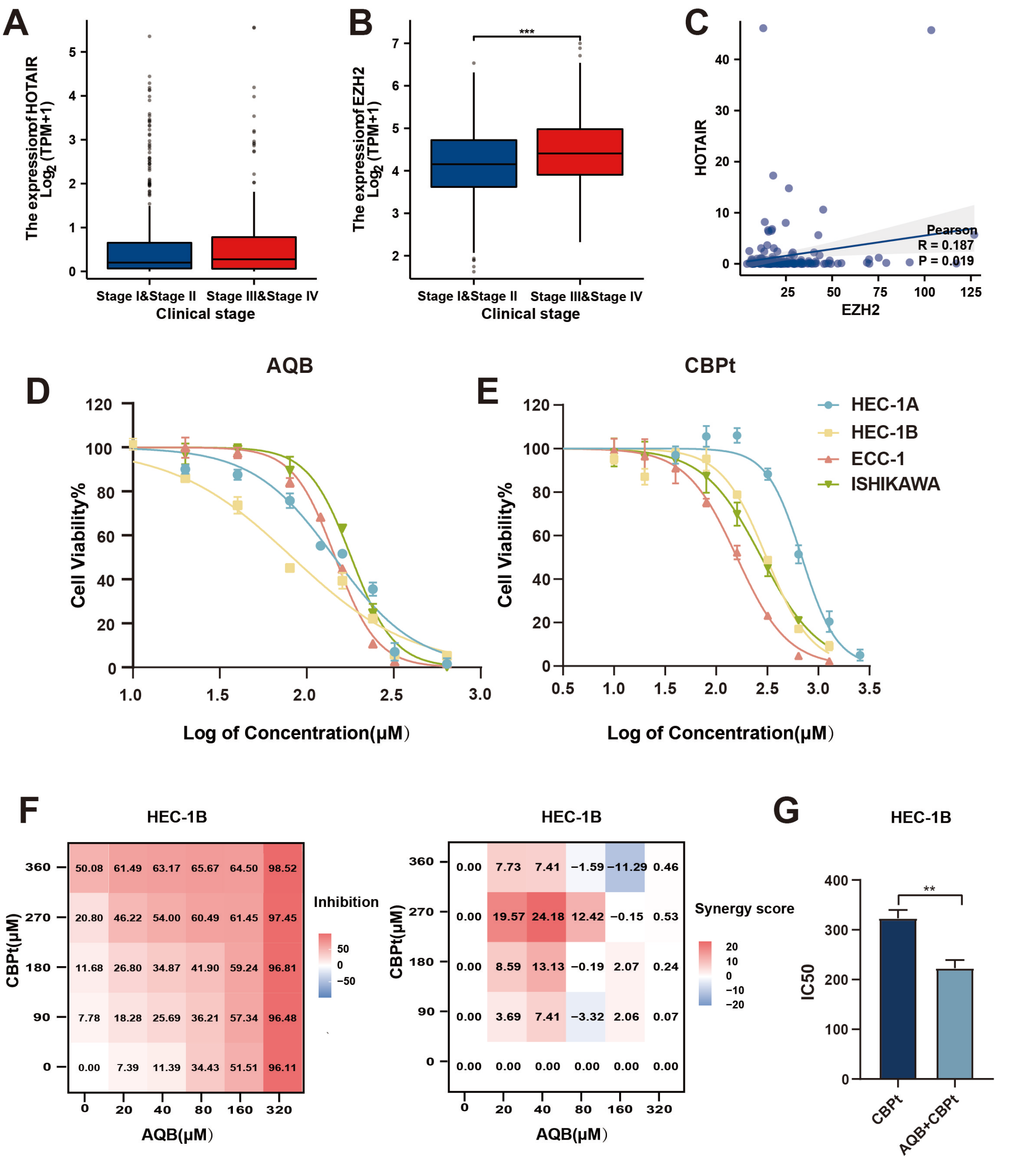

Supplementary Figure S1. (A-B) Expression levels of HOTAIR and EZH2 in EC patients grouped by clinical stages (Stage I & II vs. Stage III & IV) based on TCGA dataset. (C) Correlation analysis between HOTAIR and EZH2 expression in advanced-stage EC patients (Stage III & IV) based on TCGA dataset. (D-E) IC50 of AQB and CBPt in four EC cell lines. (F) Combination matrices of cell inhibition and synergy scores by AQB and CBPt. Data represent the mean of 3 independent experiments. (G) IC50 of CBPt after treatment with AQB. The data were expressed as the mean ± SD (n = 3). **P < 0.01, ***P < 0.001.

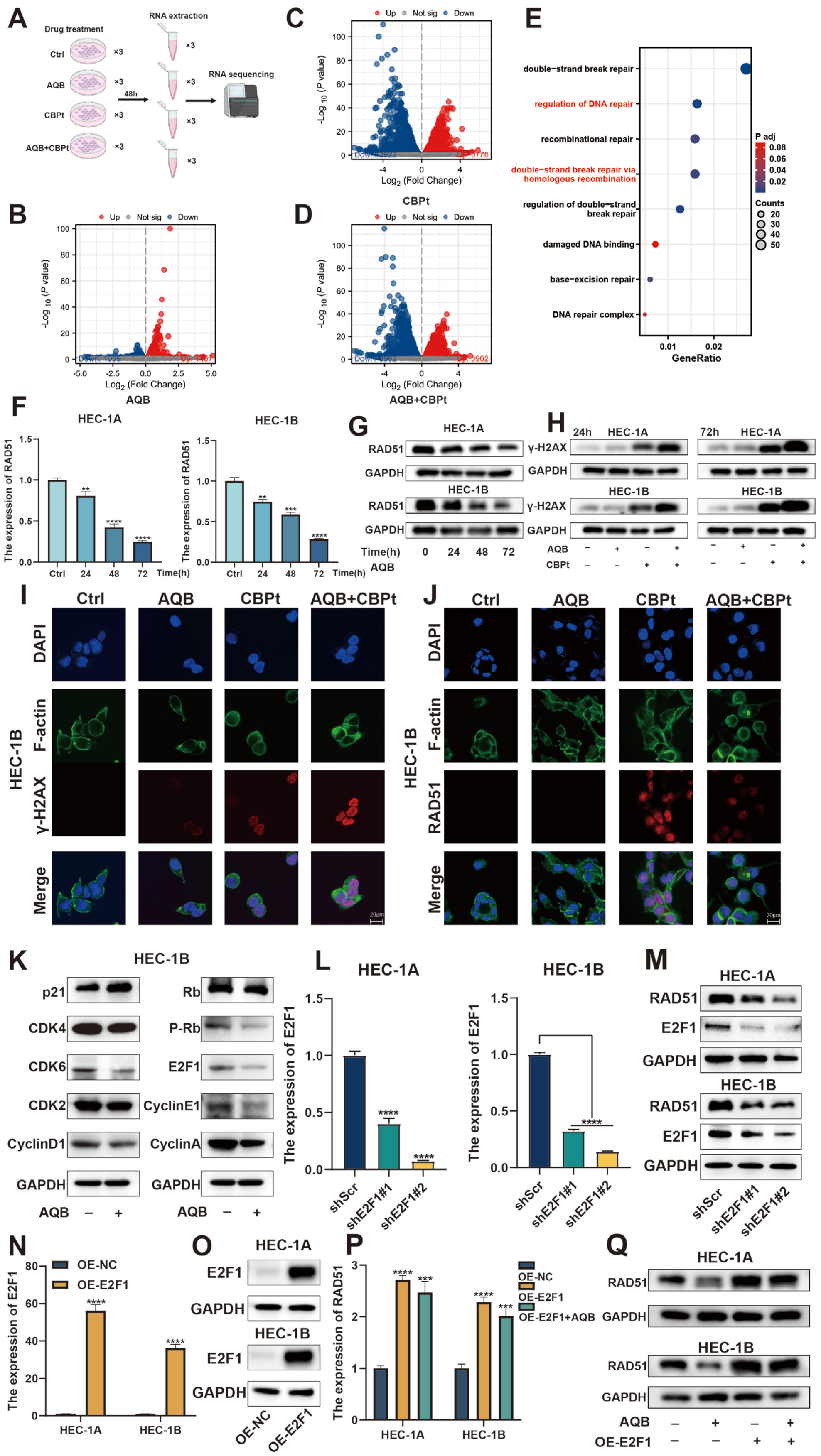

Supplementary Figure S2. (A) RNA Sequencing workflow. HEC-1B cells were treated with DMSO,

AQB (60μM), CBPt(100μM) or AQB (60μM) + CBPt(100μM) for 48h (Created with bioRender.com). Three samples per group, for a total of 12 samples, were analyzed. Volcano plot of DEGs between AQB (B), CBPt (C) or AQB+CBPt (D) and DMSO groups. (E) GO analysis of DEGs between AQB and DMSO groups. (F-G) qRT-PCR and Western blot analyses showing that AQB treatment reduced RAD51 expression in HEC-1A and HEC-1B cells at 24, 48, and 72 h. (I-J) IF images of γ-H2AX and RAD51 in HEC-1B cells after 48 h of treatment. Scale bar: 20 μm. (K)Western blotting of p21 and its downstream proteins in HEC-1B cells. (L)qRT-PCR analysis confirmed the knockdown efficiency of E2F1. (M) Western blotting was used to confirm the knockdown efficiency of E2F1 and to assess its effects on RAD51 expression in HEC-1A and HEC-1B cells. (N) qRT-PCR validation of E2F1 overexpression in HEC-1A and HEC-1B cells. (O) Western blotting confirming E2F1 overexpression in HEC-1A and HEC-1B cells. (P-Q) qRT-PCR (P) and Western blotting (Q) analyses of RAD51 expression in E2F1-overexpressing cells with or without AQB treatment. The data are expressed as the mean ± SD (n = 3). *$P < 0.05$, **$P < 0.01$, ***$P < 0.001$, ****$P < 0.0001$.

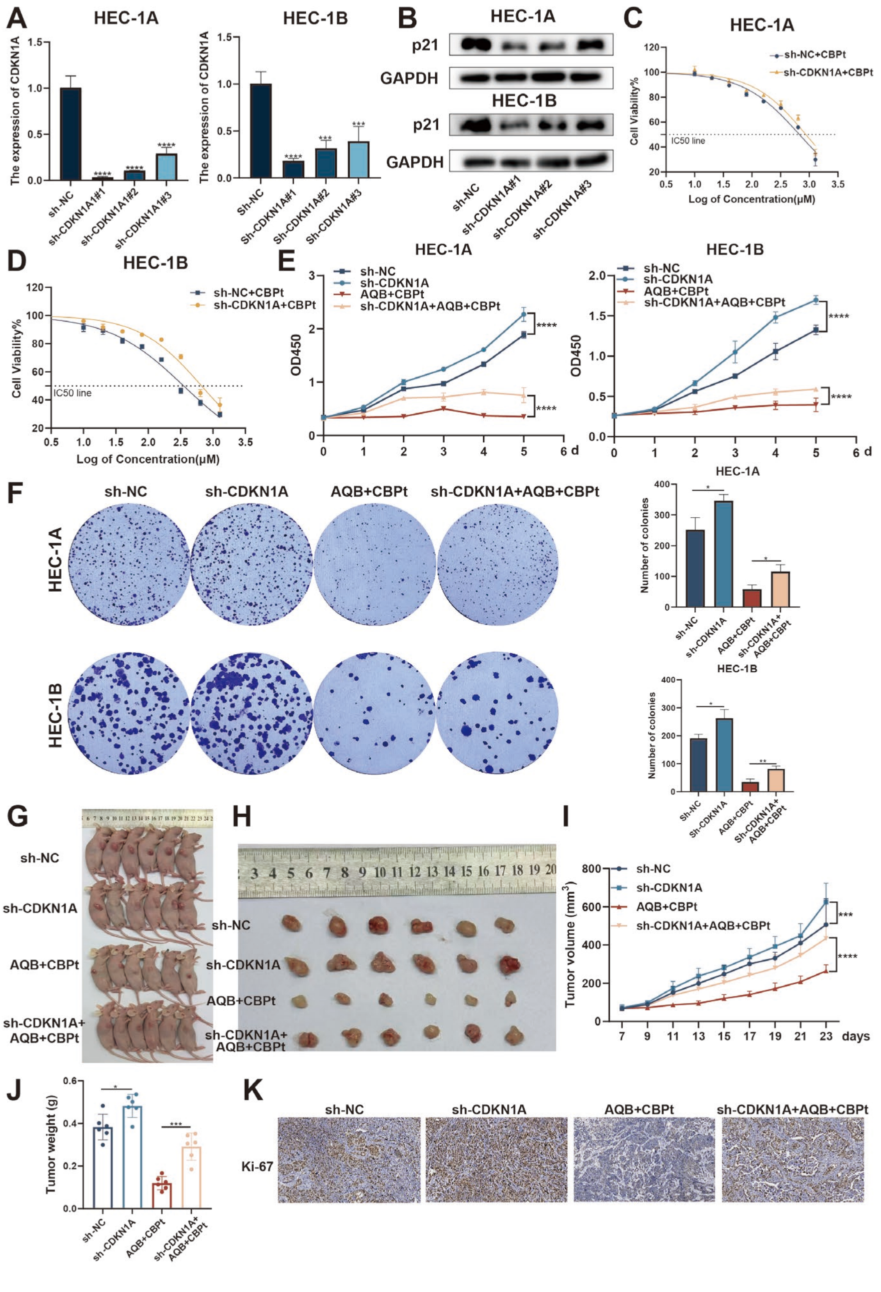

Supplementary Figure S3. (A) qRT-PCR analysis of CDKN1A knockdown efficiency in HEC-1A and HEC-1B cells. (B) Western blotting showing reduced p21 protein levels after CDKN1A knockdown. (C–D) Dose–response curves showing that CDKN1A knockdown increased CBPt IC50 in HEC-1A (674 → 873.5 μM) and HEC-1B (352.3 → 666 μM) cells. (E) CCK-8 assay showing reduced antiproliferative effect of AQB+CBPt after CDKN1A knockdown. (F) Colony formation assay showing attenuated inhibitory effect of AQB+CBPt after CDKN1A knockdown. (G–I) Subcutaneous xenograft models derived from CDKN1A-knockdown cells showing compromised response to AQB+CBPt, as reflected by representative mouse images (G), excised tumors (H), and tumor growth curves (I). (J) Tumor weights at the end of treatment. (K) Ki-67 IHC staining of xenograft tumors. Scale bars: 100 μm. Data are expressed as mean ± SD (n = 3 for cell-based assays, n = 6 nude mice). *P < 0.05, **P <0.01, ***P < 0.001, ****P < 0.0001.

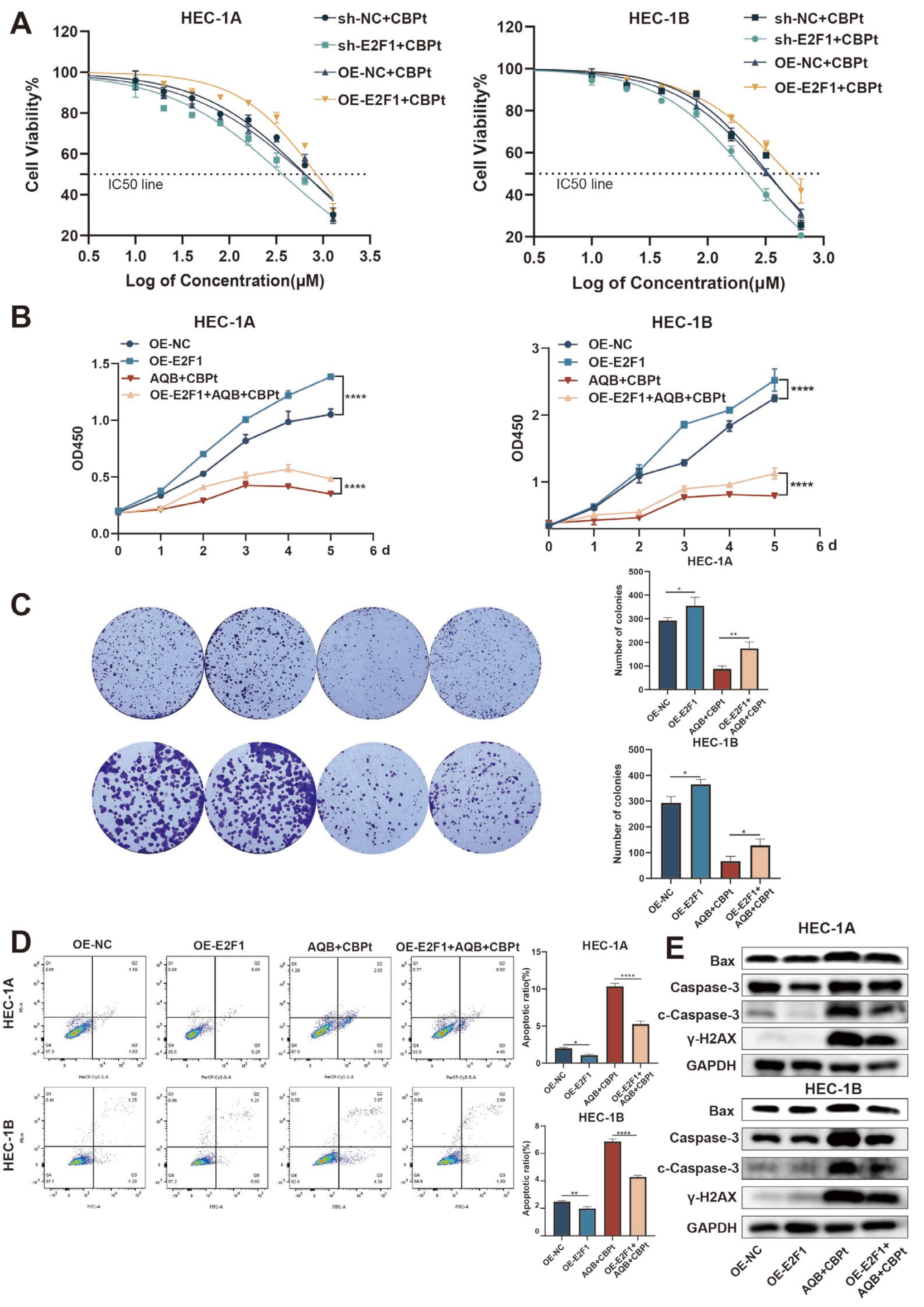

Supplementary Figure S4. (A) Dose–response curves showing that E2F1 knockdown enhanced CBPt sensitivity, reducing IC50 values (HEC-1A: 645.6 → 367.5 μM; HEC-1B: 334.6 → 221.2 μM), whereas E2F1 overexpression conferred resistance, elevating IC50 values (HEC-1A: 632.3 → 849.5 μM; HEC-1B: 323.7 → 498 μM). (B) CCK8 assay showing the effects of E2F1 overexpression on AQB+CBPt-induced growth inhibition. (C) Colony formation assay showing the effects of E2F1 overexpression on AQB+CBPt-induced reduction of clonogenic growth. (D) Flow cytometry analysis showing the effects of E2F1 overexpression on AQB+CBPt-induced apoptosis. (E) Western blotting showing the effects of E2F1 overexpression on AQB+CBPt-induced expression of Bax, caspase-3, cleaved caspase-3, and γ-H2AX. Data are expressed as mean ± SD (n = 3). *P < .05, **P < .01, ***P < 0.001, ****P < 0.0001.

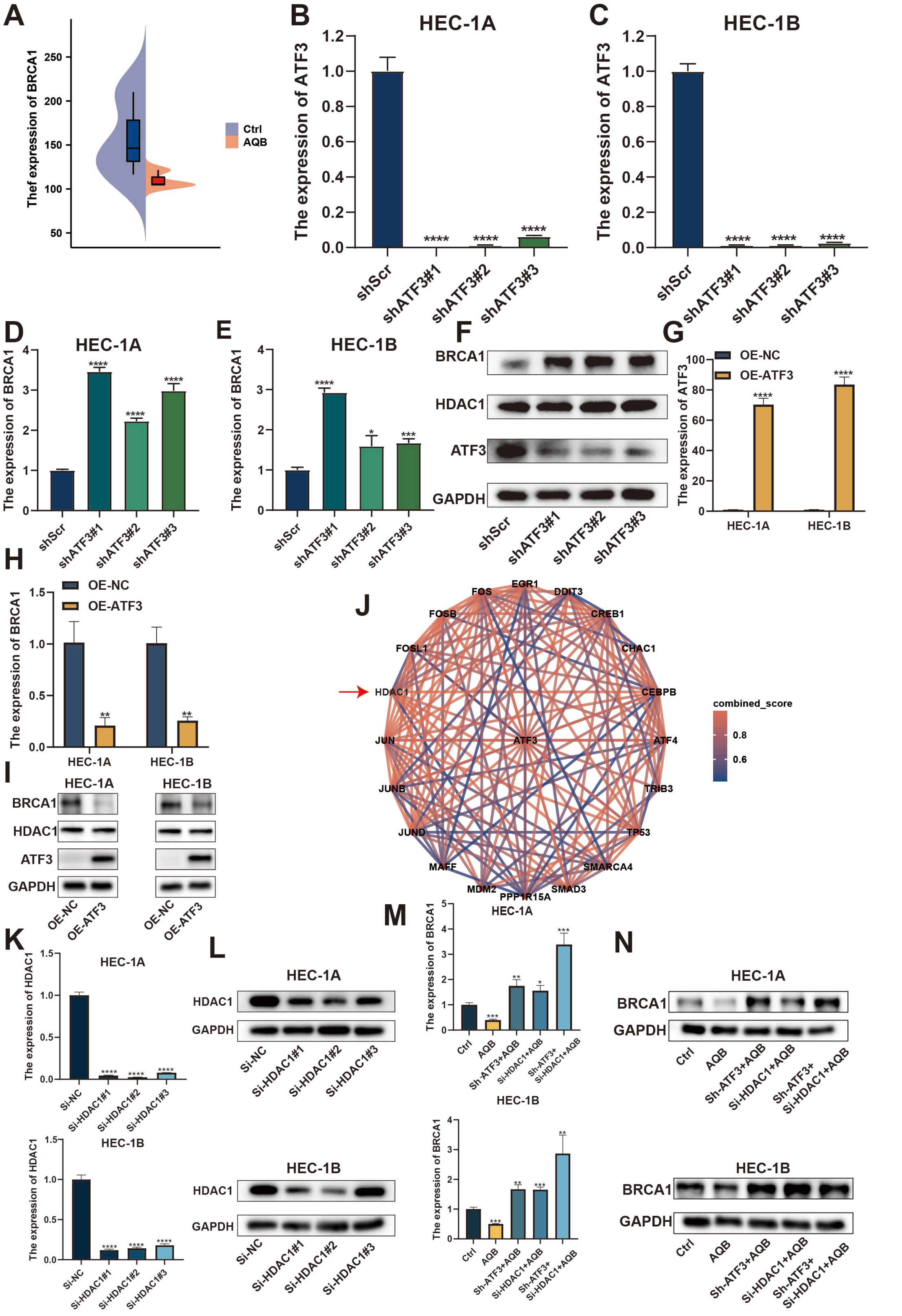

Supplementary Figure S5. (A) The RNA sequencing data indicate that AQB can downregulate the expression of BRCA1. (B-C) qRT-PCR and Western blot analyses showing that AQB treatment reduced BRCA1 expression in HEC-1A and HEC-1B cells at 24, 48, and 72 h. (D) qRT-PCR analysis confirmed

the knockdown efficiency of ATF3. (E) Expression levels of BRCA1 mRNA after ATF3 knockdown. (F)Protein expression levels of BRCA1 and HDAC1 after ATF3 knockdown in the HEC-1B cells. (G) qRT-PCR confirmed ATF3 overexpression in HEC-1A and HEC-1B cells. (H) qRT-PCR showing BRCA1 mRNA levels after ATF3 overexpression. (I) Western blotting of BRCA1, HDAC1, and ATF3 in ATF3-overexpressing cells. (J) The protein interaction map of ATF3 shows the interaction strength (combined score) and network relationship with HDAC1. (K) qRT-PCR confirmed HDAC1 knockdown using three independent siRNAs in HEC-1A and HEC-1B cells. (L) Western blotting of HDAC1 following siRNA knockdown in HEC-1A and HEC-1B cells. (M) qRT-PCR analysis of BRCA1 expression under the indicated conditions in HEC-1A and HEC-1B cells: control, AQB alone, ATF3 knockdown + AQB, HDAC1 knockdown + AQB, and combined ATF3/HDAC1 knockdown + AQB. (N) Western blotting of BRCA1 under the same conditions as in (M) for HEC-1A and HEC-1B cells. The data are expressed as mean ± SD (n = 3). *P < 0.05, **P < 0.01, ***P < 0.001, ****P < 0.0001, ns = not significant.

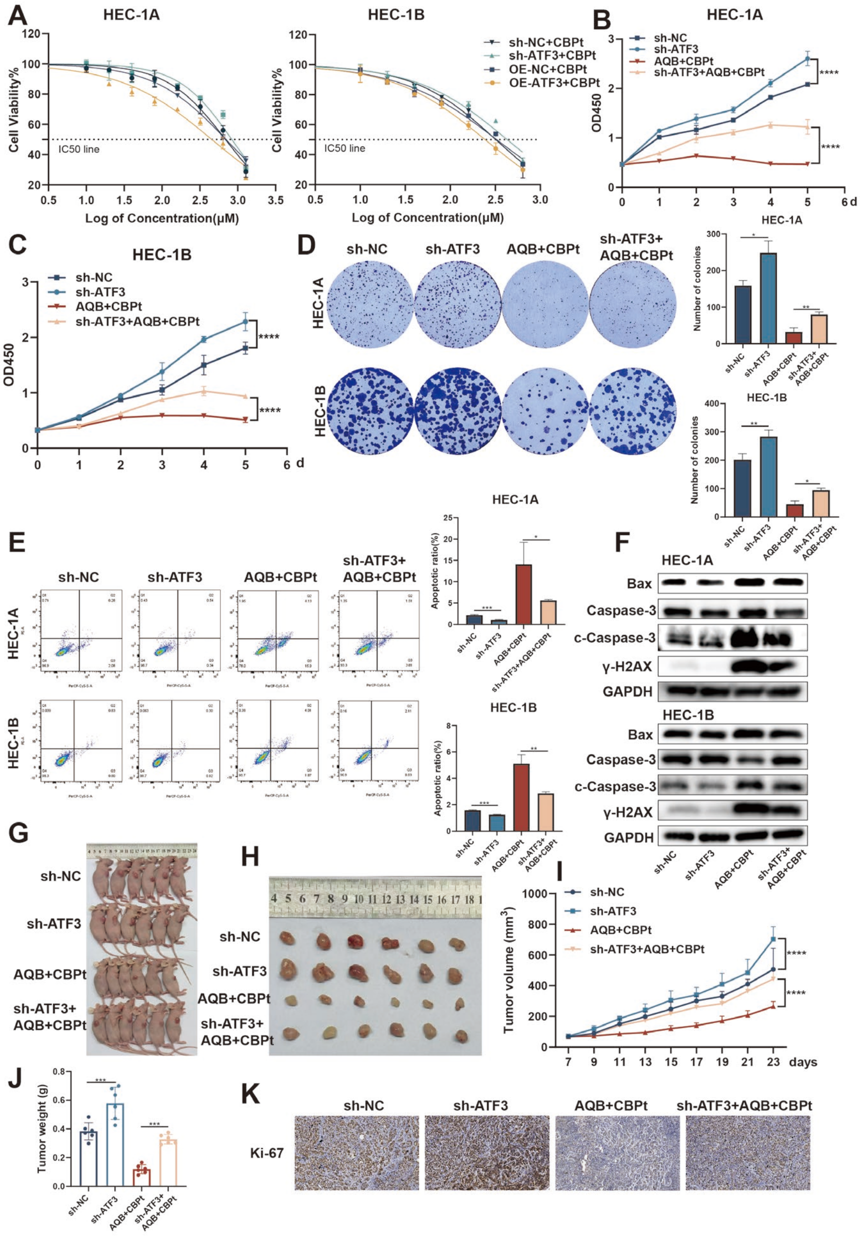

Supplementary Figure S6. (A) Dose–response curves showing that ATF3 knockdown decreased CBPt

sensitivity, increasing IC50 values (HEC-1A: 689.5 → 835.9 μM; HEC-1B: 320.3 → 428.8 μM), whereas ATF3 overexpression enhanced sensitivity, reducing IC50 values (HEC-1A: 699.6 → 455.7 μM; HEC-1B: 327.7 → 243.6 μM). (B-C) CCK8 assay showing the effects of ATF3 knockdown on AQB+CBPt-induced growth inhibition. (D) Colony formation assay showing the effects of ATF3 knockdown on AQB+CBPt-induced reduction of clonogenic growth. (E) Flow cytometry analysis showing the effects of ATF3 knockdown on AQB+CBPt-induced apoptosis. (F) Western blotting showing the effects of ATF3 knockdown on AQB+CBPt-induced expression of Bax, caspase-3, cleaved caspase-3, and γ-H2AX. (G–I) Subcutaneous xenograft models derived from ATF3-knockdown cells showing compromised response to AQB+CBPt, as reflected by representative mouse images (G), excised tumors (H), and tumor growth curves (I). (J) Tumor weights at the end of treatment. (K) Ki-67 IHC staining of xenograft tumors. Scale bars: 100 μm. Data are expressed as mean ± SD (n = 3 for cell-based assays, n = 6 nude mice). *P < 0.05, **P <0.01, ***P < 0.001, ****P < 0.0001.

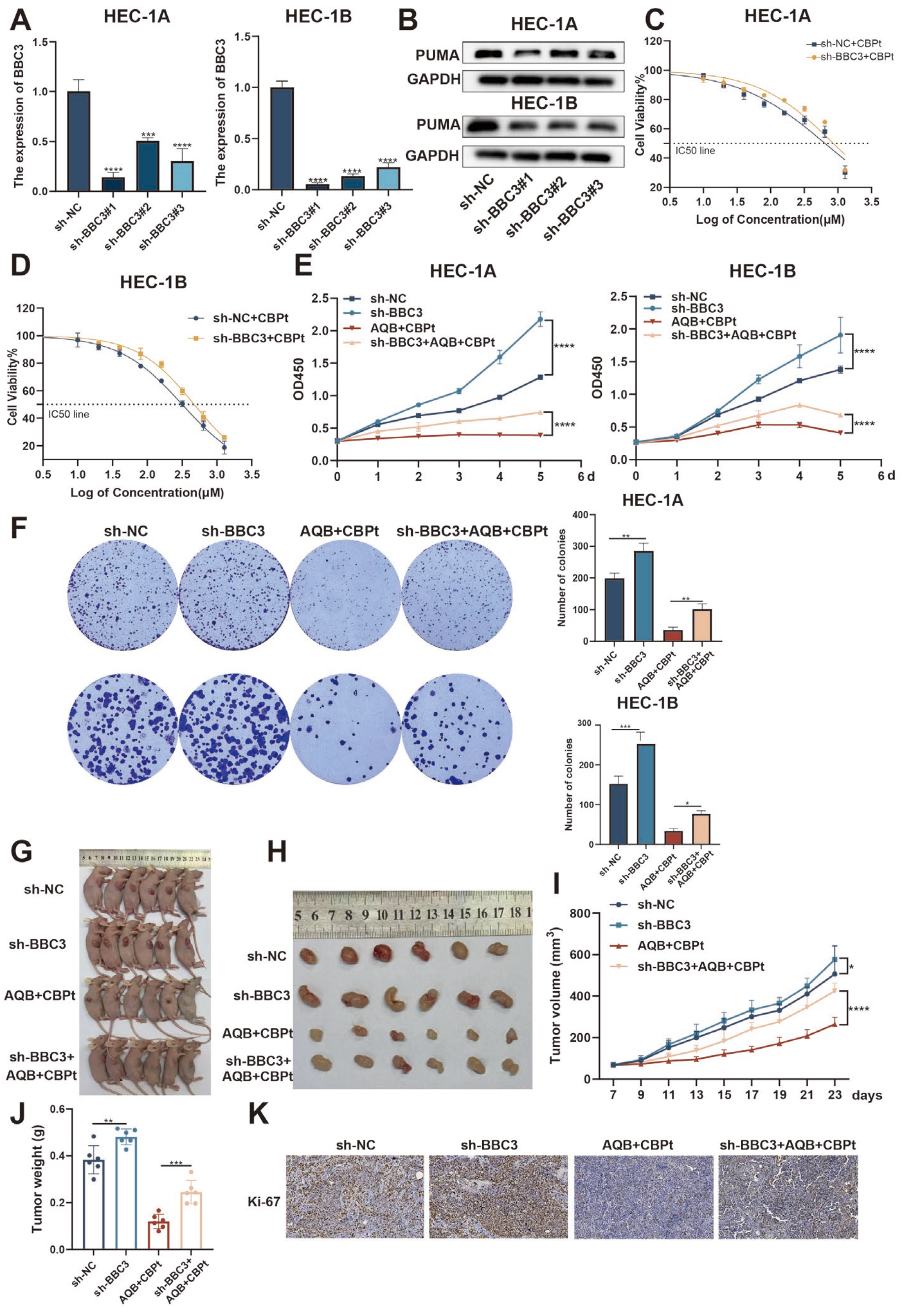

Supplementary Figure S7. (A) qRT-PCR analysis of BBC3 knockdown efficiency in HEC-1A and HEC-1B cells. (B) Western blotting showing reduced PUMA protein levels after BBC3 knockdown. (C–D) Dose–response curves showing that BBC3 knockdown increased CBPt IC50 values (HEC-1A: 643.6 → 855.4 μM; HEC-1B: 316.9 → 471.1 μM). (E) CCK-8 assay showing reduced antiproliferative effect of AQB+CBPt after BBC3 knockdown. (F) Colony formation assay showing attenuated inhibitory effect of AQB+CBPt after BBC3 knockdown. (G–I) Subcutaneous xenograft models derived from BBC3-knockdown cells showing compromised response to AQB+CBPt, as reflected by representative mouse images (G), excised tumors (H), and tumor growth curves (I). (J) Tumor weights at the end of treatment. (K) Ki-67 IHC staining of xenograft tumors. Scale bars: 100 μm. Data are expressed as mean ± SD (n = 3 for cell-based assays, n = 6 nude mice). *P < 0.05, **P <0.01, ***P < 0.001, ****P < 0.0001.

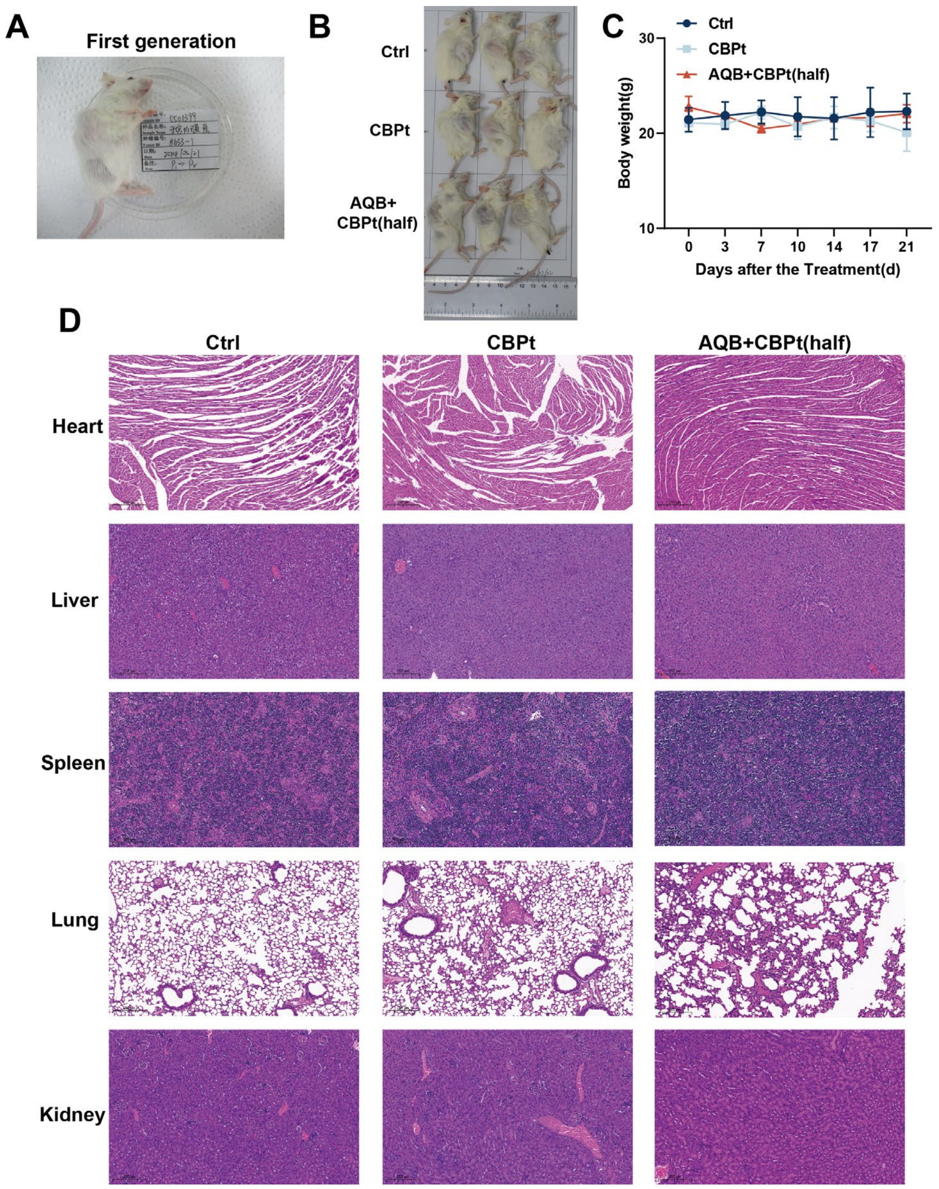

Supplementary Figure S8. (A) First-generation PDX mouse xenograft model. (B) Tumor-bearing mice images. (C) Body weight changes in the three groups of mice. (D) H&E staining of major target organs dissected from mice at the end of experiment. Scale bar: 200 μm.

Supplementary Table S1.

**Primers**	
**shRNA oligos**	
shE2F1#1-F	GGACUCUUCGGAGAACUUUTT
shE2F1#1-R	AAAGUUCUCCGAAGAGUCCTT
shE2F1#2-F	GCUGGACCACCUGAUGAAUTT
shE2F1#2-R	AUUCAUCAGGUGGUCCAGCTT
shATF3#1-F	GGCGACGAGAAAGAAATAAGA
shATF3#1-R	TCTTATTTCTTTCTCGTCGCC
shATF3#2-F	GAAGCTGGAAAGTGTGAATGC
shATF3#2-R	GCATTCACACTTTCCAGCTTC
shATF3#3-F	GCCTTAACACACTGGCCATTC
shATF3#3-R	GAATGGCCAGTGTGTTAAGGC
sh-CDKN1A#1	CGCTCTACATCTTCTGCCTTA
sh-CDKN1A#2	GACAGCAGAGGAAGACCATGT
sh-CDKN1A#3	AAGACCATGTGGACCTGTCAC
sh-BBC3#1	GAAGAGCAAATGAGCCAAACG
sh-BBC3#2	ACGTGTGACCACTGGCATTCA
sh-BBC3#3	GCCCAATTAGGTGCCTGCACC
**siRNA oligos**	
si-HDAC1#1-F	GGAUUGAUGACGAGUCCUATT
si-HDAC1#1-R	UAGGACUCGUCAUCAAUCCTT
si-HDAC1#2-F	GGAGAAGAUCAAACAGCGATT
si-HDAC1#2-R	UCGCUGUUUGAUCUUCUCCTT
si-HDAC1#3-F	GCUCCAUCCGUCCAGAUAATT
si-HDAC1#3-R	UUAUCUGGACGGAUGGAGCTT
**qRT-PCR Primers**	
HOTAIR-F	CCAGAGAACGCTGGAAAAACCTG
HOTAIR-R	GGAGATGATAAGAAGAGCAAGGAA

EZH2-F	GACCTCTGTCTTACTTGTGGAGC
EZH2-R	CGTCAGATGGTGCCAGCAATAG
MRE11-F	CAGCAACCAACAAAGGAAGAGGC
MRE11-R	GAGTTCCTGCTACGGGTAGAAG
RAD50-F	GGAAGAGCAGTTGTCCAGTTACG
RAD50-R	GAGTAAACTGCTGTGGCTCCAG
CHEK1-F	GTGTCAGAGTCTCCCAGTGGAT
CHEK1-R	GTTCTGGCTGAGAACTGGAGTAC
CHEK2-F	GACCAAGAACCTGAGGAGCCTA
CHEK2-R	GGATCAGATGACAGCAGGAGTTC
RAD51-F	TCTCTGGCAGTGATGTCCTGGA
RAD51-R	TAAAGGGCGGTGGCACTGTCTA
E2F1-F	GTGTCAGGACCTTCGTAGCA
E2F1-R	TCGGGCCTTGTTTGCTCTTA
ATF3-F	CGCTGGAATCAGTCACTGTCAG
ATF3-R	CTTGTTTCGGCACTTTGCAGCTG
BRCA1-F	GAAACCGTGCCAAAAGACTTC
BRCA1-R	CCAAGGTTAGAGAGTTGGACAC
HDAC1-F	GGATTGATGACGAGTCCTATGAG
HDAC1-R	AGTCTGAGCCACACTGTAAGACC
BBC3-F	GAAAGGCTGTTGTGCTGGTG
BBC3-R	AGGCTAGTGGTCACGTTTGG
GAPDH-F	GGTGGTCTCCTCTGACTTCAACA
GAPDH-R	GTTGCTGTAGCCAAATTCGTTGT
**ChIP-qPCR Primers**	
RAD51-primer-F	GGCGAAAACACAAGTGGACC
RAD51-primer-R	CCGCTCTGATCTCGGACTTC
BRCA1-primer-F	TGTGCCCGCTCTGGTATTG

BRCA1-primer-R	CGGAATGAAAGGTCTTCGCC
BBC3-primer-F	TGGTTTACTCATGACCCACAGTT
BBC3-primer-R	CTCTTTCGCTTTCCCCGATT

Supplementary Table S2.

**Antibodies**			
**Name**	**Company**	**Cat**	**Application**
γ-H2AX	Cell Signaling Technology	9718	WB, IF
RAD50	Cell Signaling Technology	3427T	WB
MRE11	Cell Signaling Technology	4847T	WB
CHK1	Affinity Biosciences	AF6004	WB
p-CHK1(ser345)	Cell Signaling Technology	2348	WB
p-CHK1(ser296)	Cell Signaling Technology	90178	WB
CHK2	Affinity Biosciences	AF6033	WB
p-CHK2	Cell Signaling Technology	82263	WB
E2F1	Cell Signaling Technology	3742	WB, ChIP
RAD51	abcam	ab133534	WB, IF, IHC
p21	Cell Signaling Technology	2947	WB
CDK4	Cell Signaling Technology	12790	WB
CDK6	Cell Signaling Technology	13331	WB
CyclinD1	Cell Signaling Technology	2978	WB
CyclinE1	Cell Signaling Technology	4129	WB
CDK2	Cell Signaling Technology	18048	WB
Rb	Affinity Biosciences	DF6840	WB
p-Rb	Cell Signaling Technology	9307	WB
CyclinA	Cell Signaling Technology	67955	WB
BRCA1	Cell Signaling Technology	50799	WB, IHC
ATF3	Cell Signaling Technology	18665	WB, ChIP, IP

HDAC1	Cell Signaling Technology	34589	WB, ChIP
HDAC1	Proteintech	66085-1-Ig	WB
H3K27ac	Cell Signaling Technology	8173	ChIP
H3K27me3	Cell Signaling Technology	9733	ChIP
PUMA	Proteintech	55120-1-AP	WB
Bax	Cell Signaling Technology	5023T	WB
Caspase-3	Cell Signaling Technology	9662	WB
Cleaved Caspase-3	Cell Signaling Technology	9661	WB, IF
Ki-67	Cell Signaling Technology	9449	IHC
GAPDH	Proteintech	60004-1-Ig	WB

Supplementary Table S3.

Bone Marrow Suppression Incidence and Severity Across Different Chemotherapy Cycles

Character	n (%)
Total number of patients	127(100%)
Total cycles assessed	500(100%)*
Myelosuppression rate	356(71.2%)
Grade 1	208(41.6%)
Grade 2	118(23.6%)
Grade 3-4	30(6%)
**Chemotherapy Cycles**	
**Short Regimen (1–4 cycles)**	
Myelosuppression rate	281(68.2%)
Moderate to Severe Myelosuppression (≥ Grade 2)	115(27.9%)
**long Regimen (5–8 cycles)**	
Myelosuppression rate	75(85.2%)
Moderate to Severe Myelosuppression (≥ Grade 2)	33(37.5%)

Note: Grading of myelosuppression is based on Common Terminology Criteria for Adverse Events (CTCAE) criteria.; Short regimen: chemotherapy cycles 1-4; Long regimen: cycles 5-8; Total number of chemotherapy cycles: 536; Age: 60.62 ± 10.04 years.

Supplementary Table S4.

Blood routine analysis in blood samples of mice after 7 days treatment.

Group	NEU (%)	RBC (10^6/μL)	HGB (g/dL)	HCT (%)	RDW (%)	PDW (%)
**Ctrl**	**62.27±3.72**	**2.83 ± 0.75**	**45.33±13.05**	**18.10±5.00**	**16.40±0.26**	**43.07±1.66**
**CBPt**	**74.43±1.78**	**2.93 ± 0.23**	**43.33±2.89**	**16.87±1.29**	**16.58±0.32**	**50.47±3.82**
**AQB+CBPt(half)**	**72.97±17.55**	**2.37 ± 0.15**	**38.00±2.65**	**0.63 ± 0.15**	**16.47±0.21**	**43.40±3.21**

Blood routine analysis in blood samples of mice after 14 days treatment.

Group	NEU (%)	RBC (10^6/μL)	HGB (g/dL)	HCT (%)	RDW (%)	PDW (%)
**Ctrl**	**65.60±2.00**	**5.90± 2.30**	**95.67±32.58**	**37.80±13.90**	**16.20±0.80**	**38.00±3.90**
**CBPt**	**72.50±5.30**	**4.80±2.30**	**79.33 ±38.70**	**30.70±14.50**	**17.80±1.90**	**46.10±7.08**
**AQB+CBPt(half)**	**69.00±0.60**	**6.70±2.50**	**109.33±2.65**	**42.20 ±13.00**	**16.80±1.60**	**37.20±0.21**

Blood routine analysis in blood samples of mice after 21 days treatment.

Group	NEU (%)	RBC (10^6/μL)	HGB (g/dL)	HCT (%)	RDW (%)	PDW (%)
**Ctrl**	**71.27±4.15**	**3.97± 1.76**	**65.67±29.02**	**24.7±11.52**	**18.40±0.70**	**38.80±3.94**
**CBPt**	**71.87±4.15**	**3.83±0.12**	**63.67±3.06**	**25.33±1.91**	**18.97±1.36**	**43.97±1.26**
**AQB+CBPt(half)**	**74.37±8.75**	**3.70±0.00**	**63.00±2.00**	**25.37±1.76**	**19.57±1.10**	**41.17±3.95**

Supplementary Table S5.

Values of serum enzymes in blood samples of mice after 21 days continuous treatment.

Group	ALT (U/L)	AST (U/L)	T-Bil (μmol/L)	D-Bil (μmol/L)	ALB (g/L)	BUN (mmol/L)	CREA (μmol/L)
Ctrl	37.00±15.13	301.33±59.90	0.83±0.06	0.10±0.17	46.17±2.32	9.70±0.78	20.00±1.00
CBPt	37.67±5.13	291.67±43.43	0.87±0.15	0.13±0.23	45.83±2.93	10.63±1.08	21.33±1.53
AQB+CBPt(half)	35.00±6.24	308.00±37.72	0.87±0.32	0.23±0.21	46.27±3.40	9.73±0.42	21.67±3.06
