# Supplementary material for: AQB improves carboplatin sensitivity in endometrial cancer through dual DNA repair modulation: suppression of the p21-E2F1-RAD51 and ATF3-HDAC1-BRCA1 signaling
Source: Cell Death Dis. 2025 Dec 6;17(1):70. doi: 10.1038/s41419-025-08287-4 (PMC12828040; doi:10.1038/s41419-025-08287-4)
Supplement: Supplementary file 2 — Western blot data. [file 41419_2025_8287_MOESM2_ESM.docx]

Figure 2 C


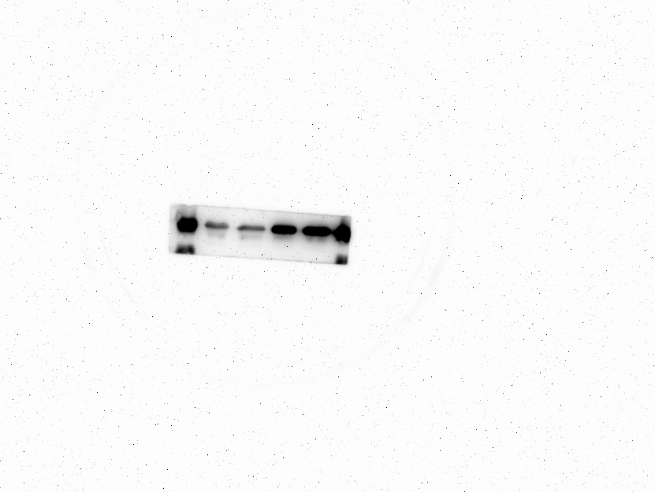

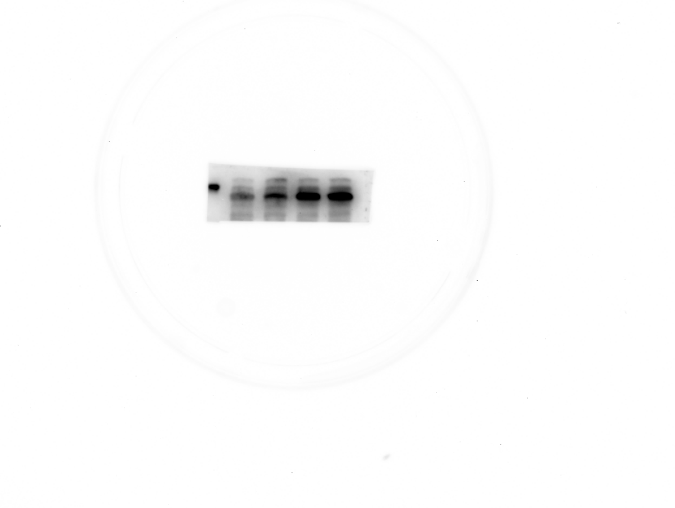
HEC-1A γ-H2AX HEC-1B γ-H2AX


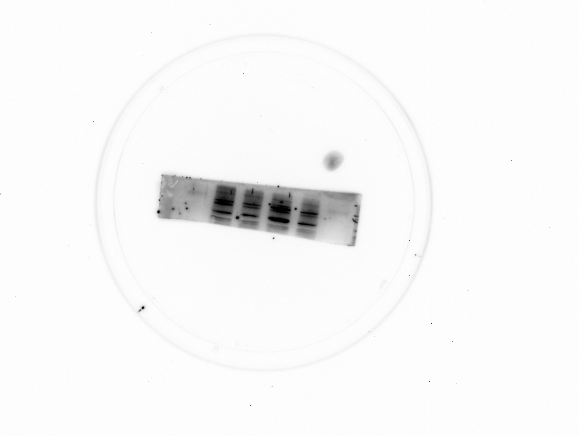

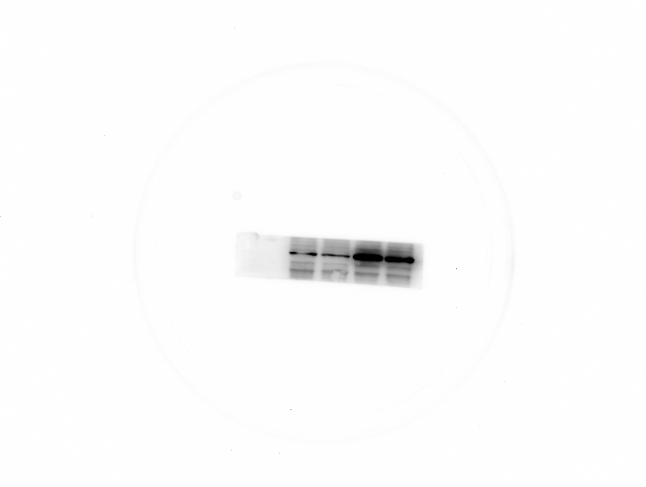

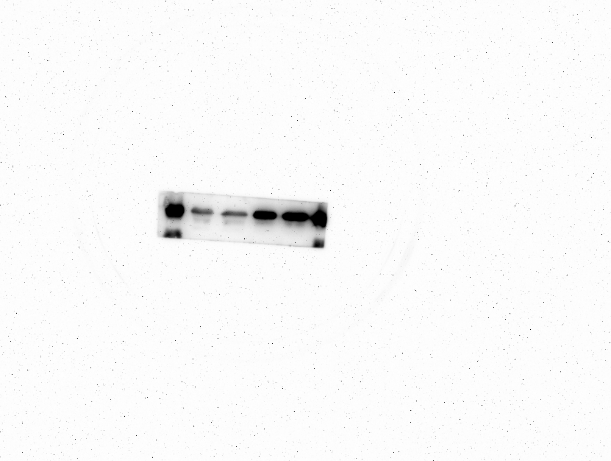

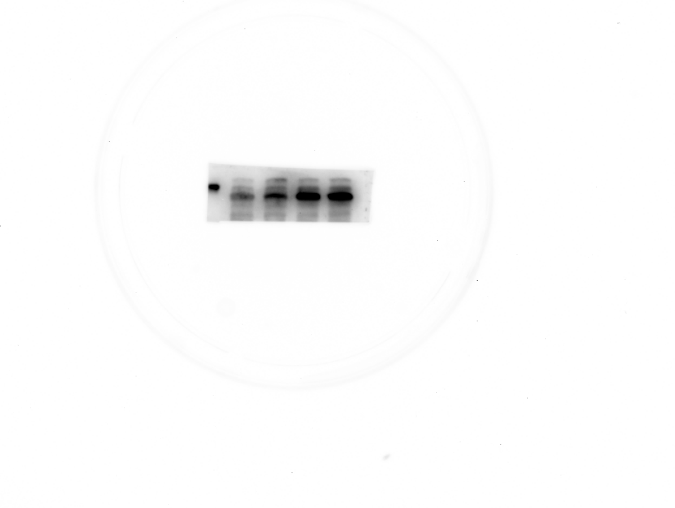
HEC-1A RAD50 HEC-1B RAD50


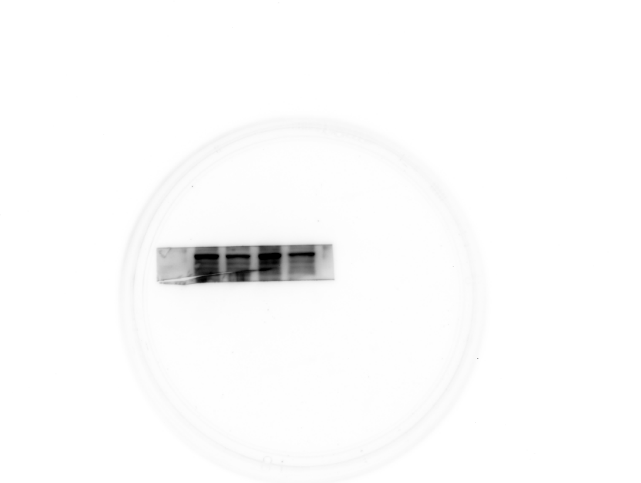

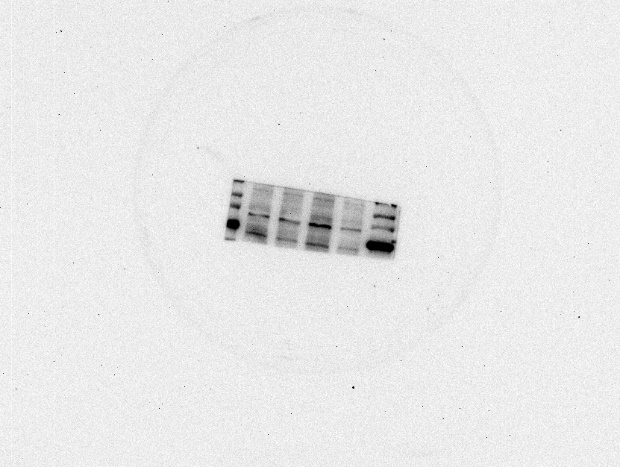
HEC-1A MRE11 HEC-1B MRE11


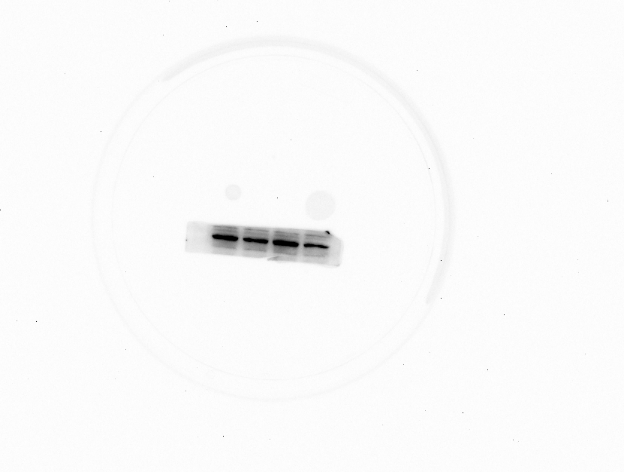

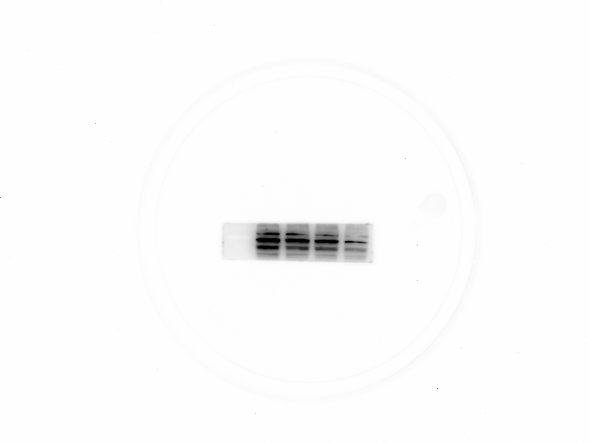
HEC-1A CHK1 HEC-1B CHK1


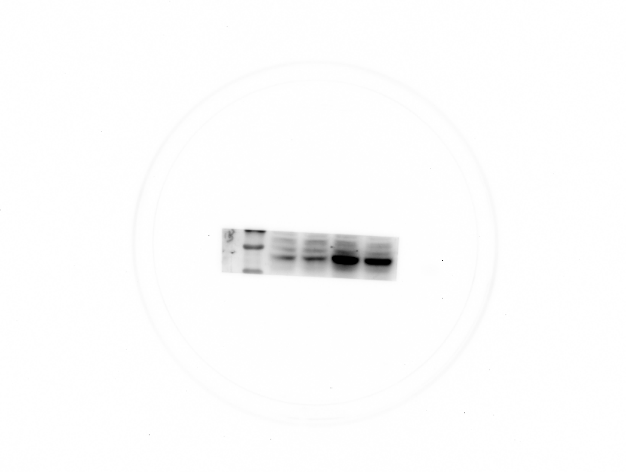

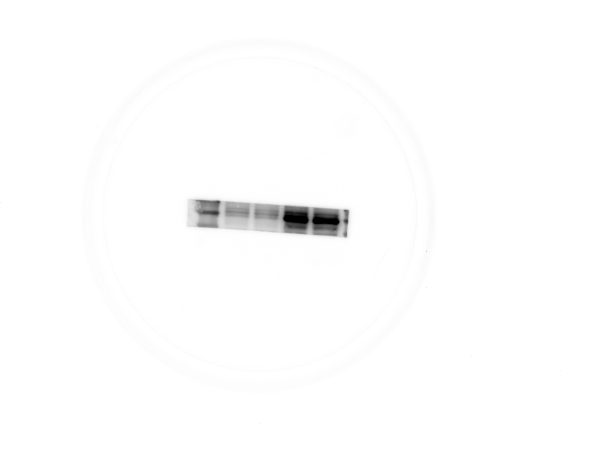
HEC-1A p-CHK1(345) HEC-1B p-CHK1(345)


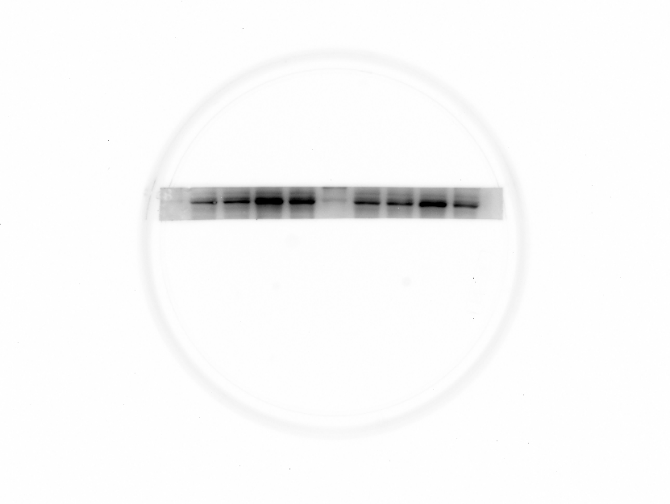

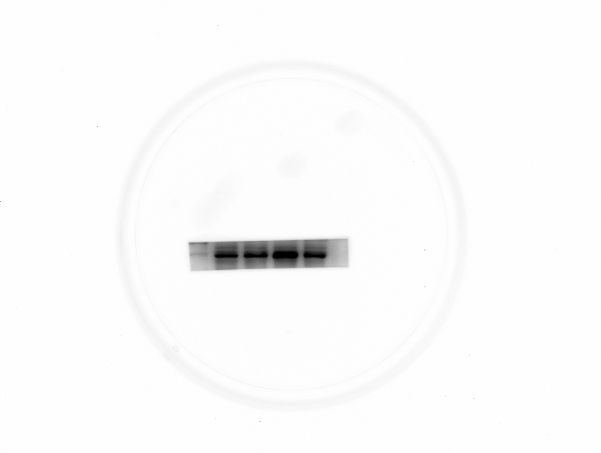
HEC-1A p-CHK1(296) HEC-1B p-CHK1(296)

HEC-1A CHK2 HEC-1B CHK2


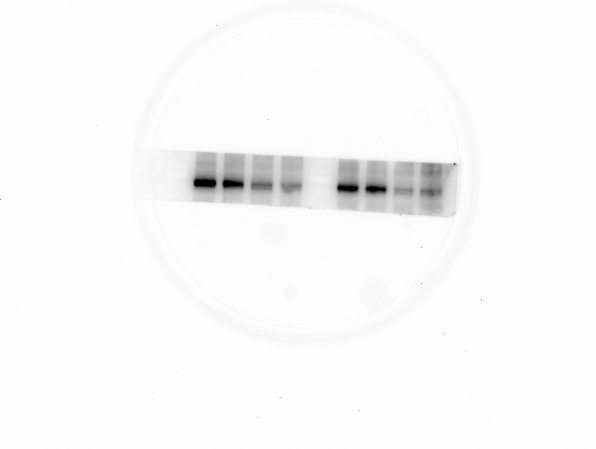

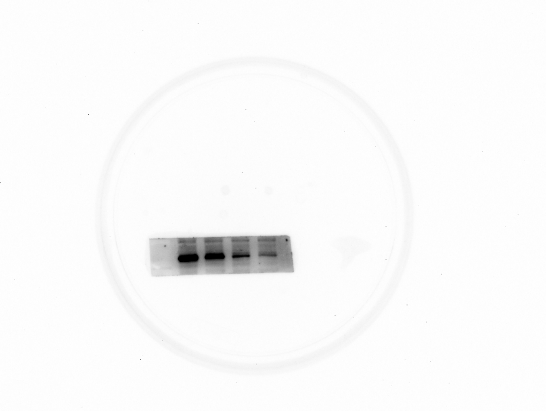


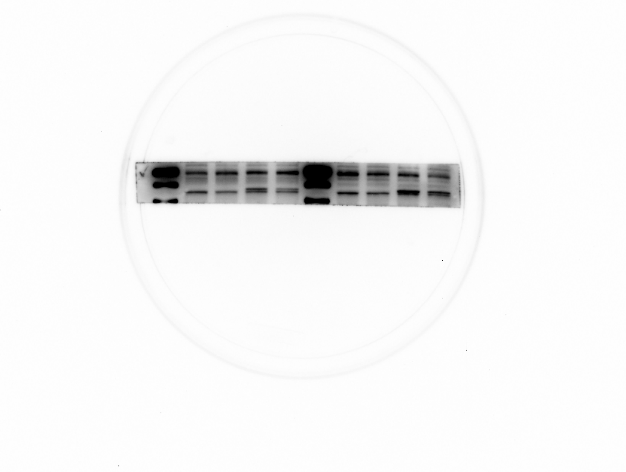

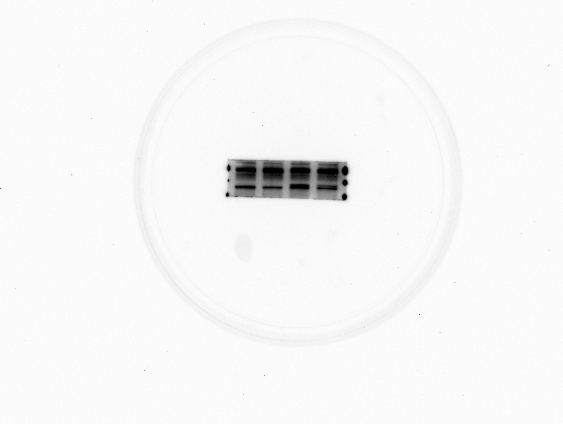
HEC-1A p-CHK2 HEC-1B p-CHK2


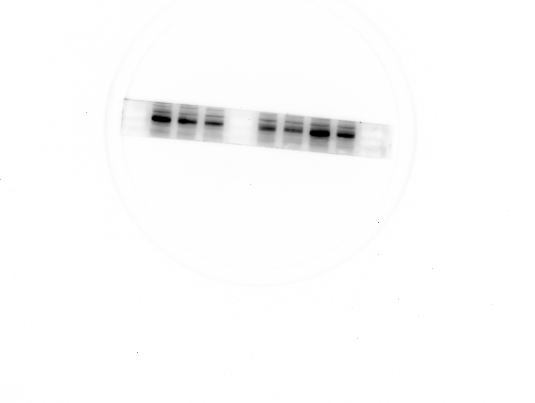

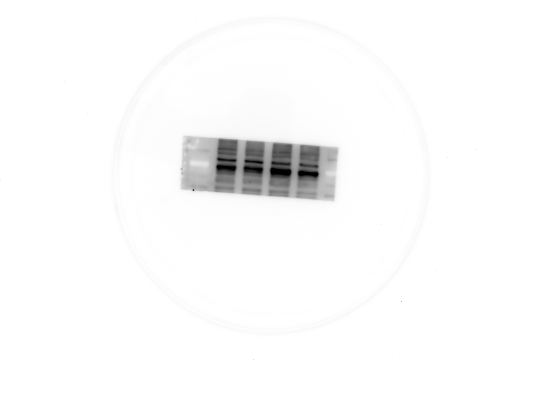
HEC-1A E2F1 HEC-1B E2F1


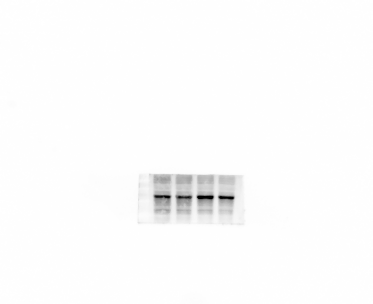

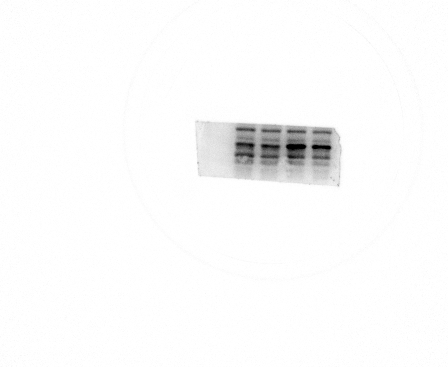
HEC-1A RAD51 HEC-1B RAD51


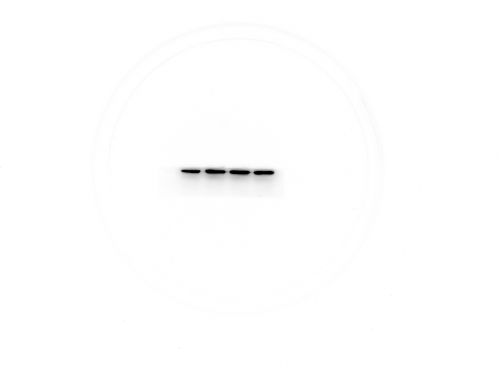

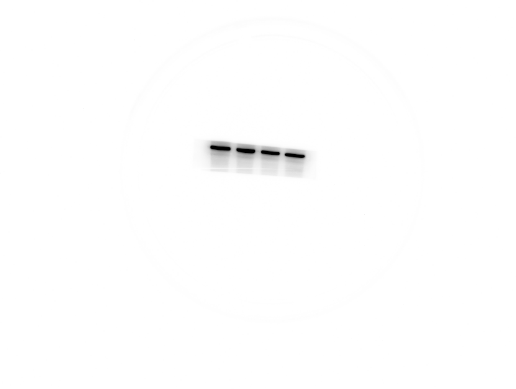
HEC-1A GAPDH HEC-1B GAPDH


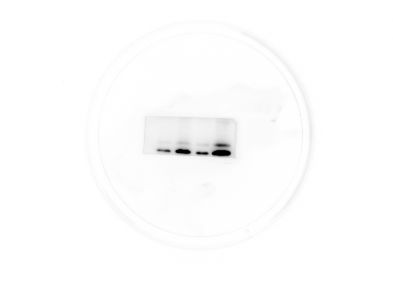

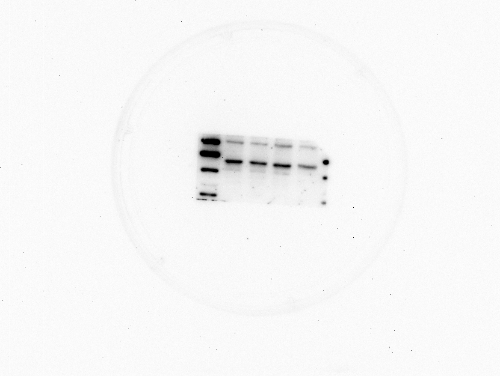

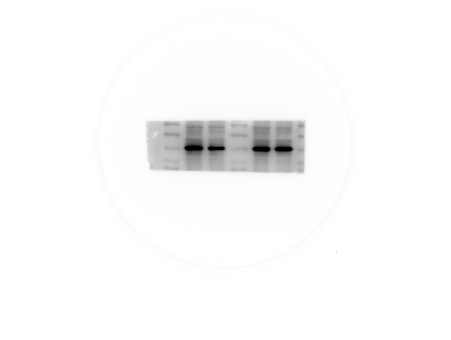
Figure 2I HEC-1A P21 CDK4 CDK6


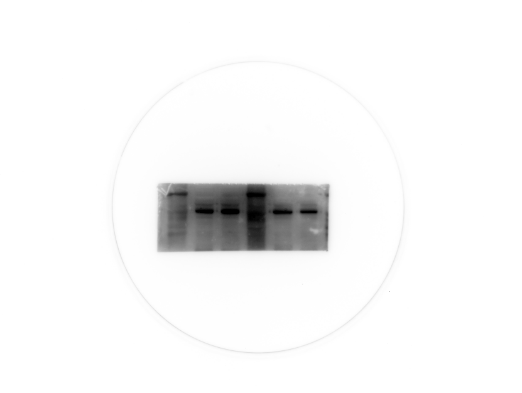




CDK2 CyclinD1 GAPDH


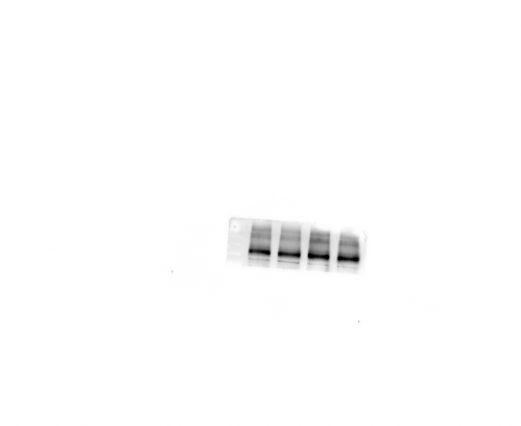

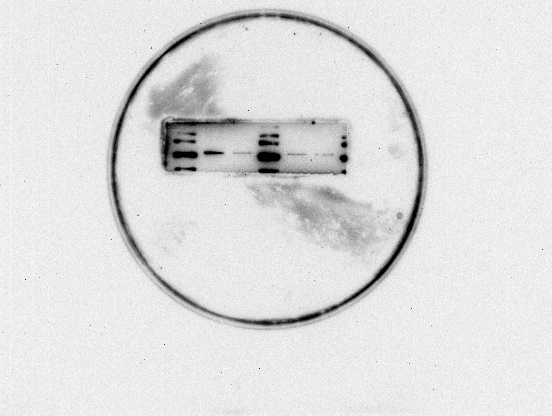
Rb p-Rb E2F1


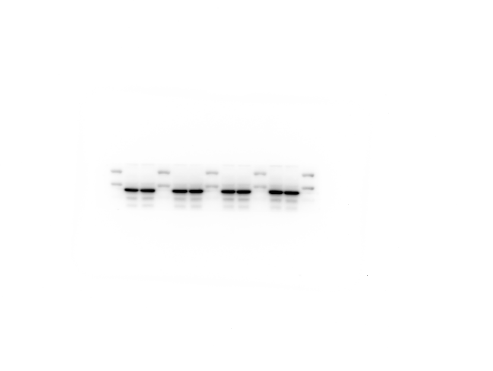

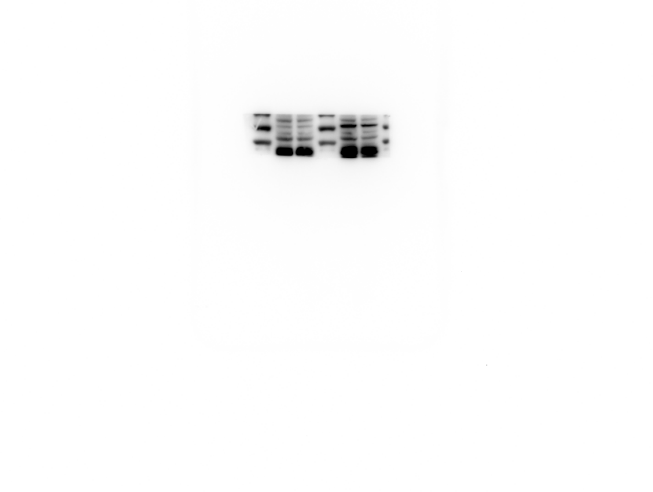


C
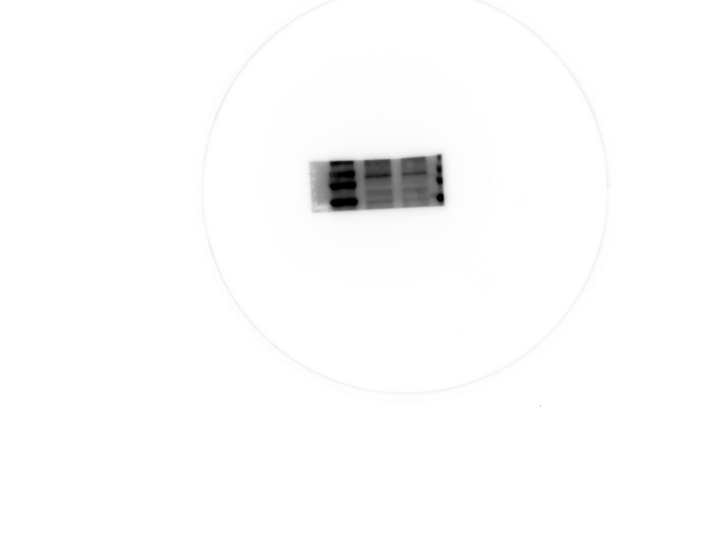
yclinE1 CyclinA GAPDH

Figure 3D


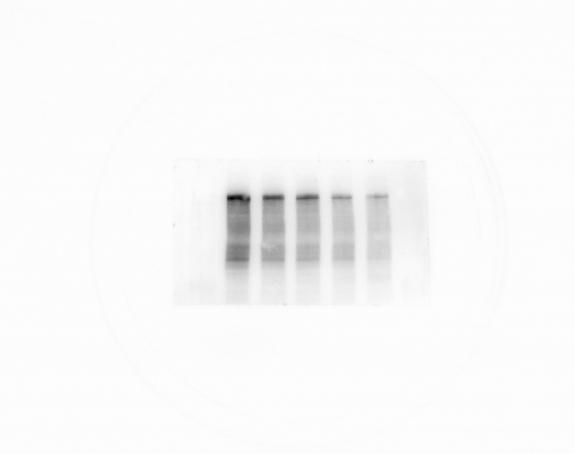


HEC-1A BRCA1 HEC-1B BRCA1


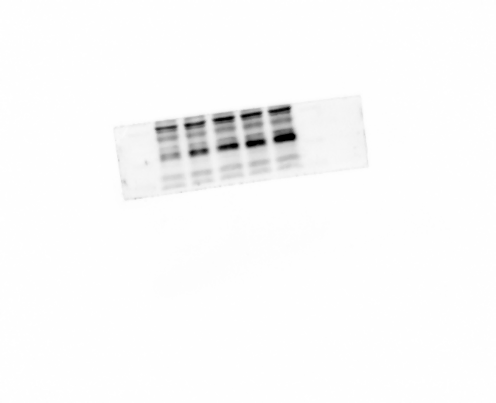

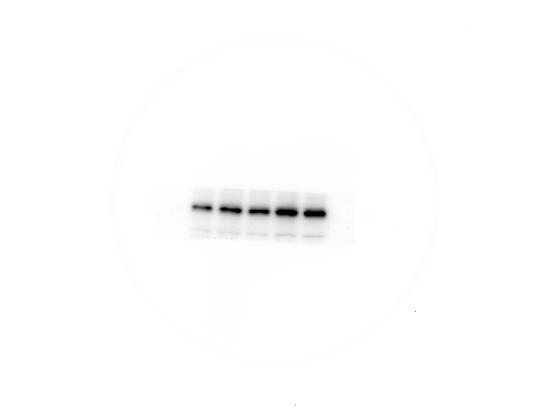
HEC-1A ATF3 HEC-1B ATF3


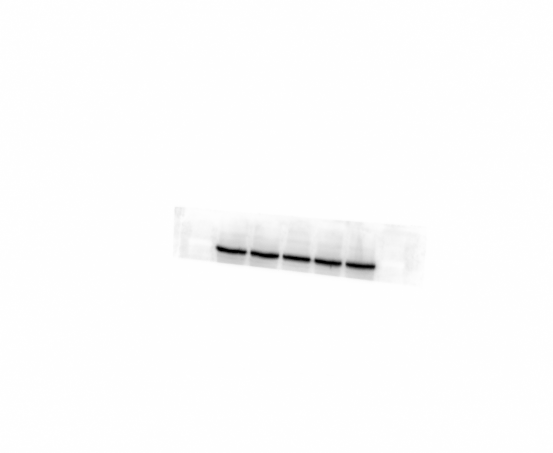


HEC-1A HDAC1 HEC-1B HDAC1




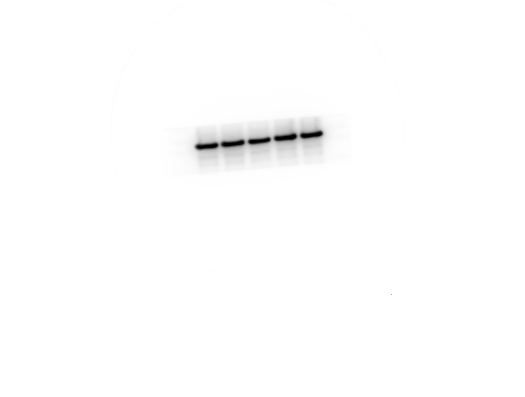
HEC-1A GAPDH HEC-1B GAPDH

Figure 3F


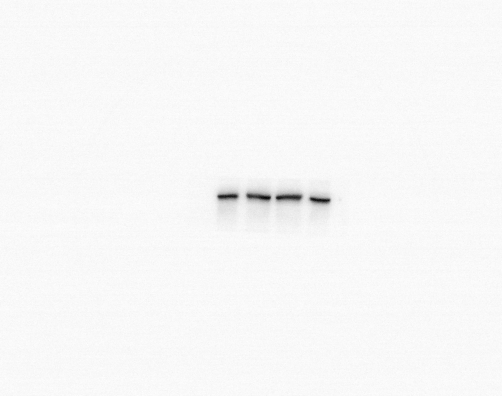

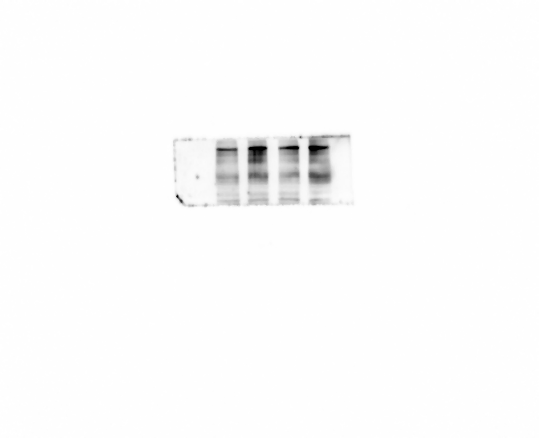
HEC-1A BRCA1 HDAC1


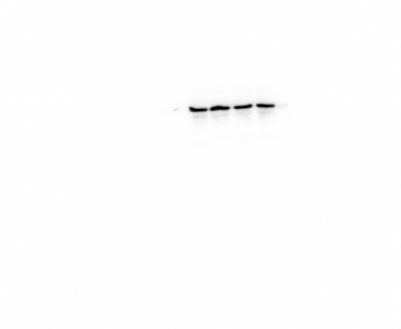

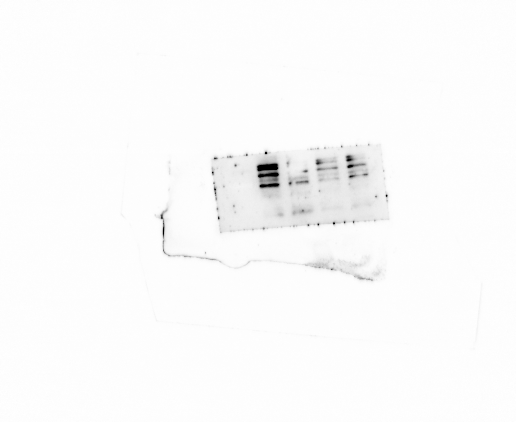
HEC-1A ATF3 GAPDH


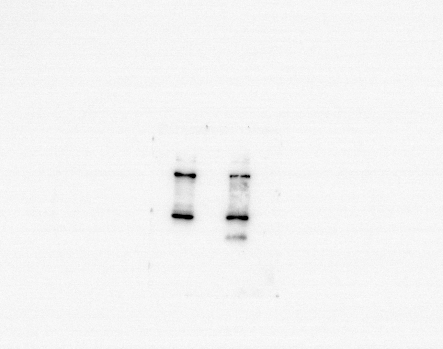

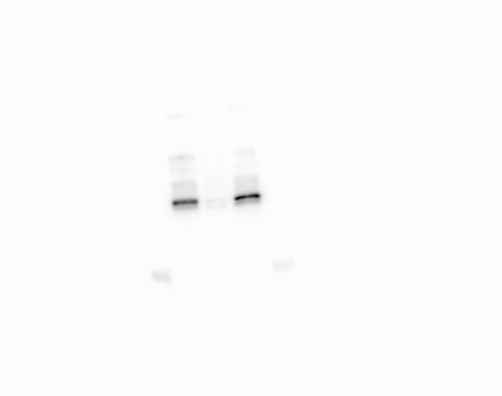
Figure 3H HEC-1A HDAC1 ATF3


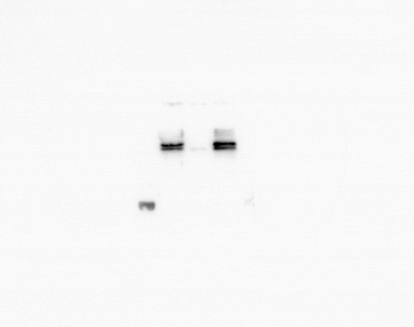

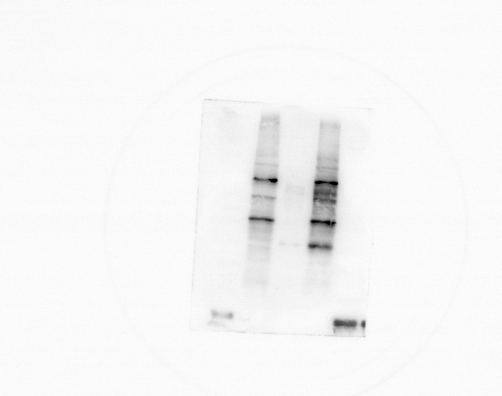
 HEC-1B HDAC1 ATF3

Figure 4D


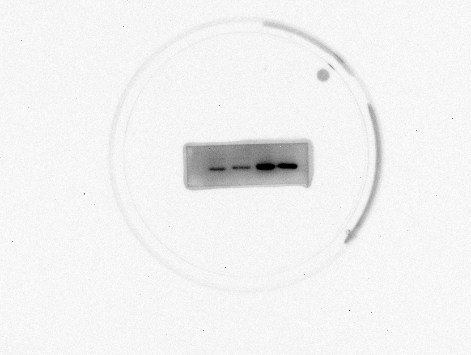

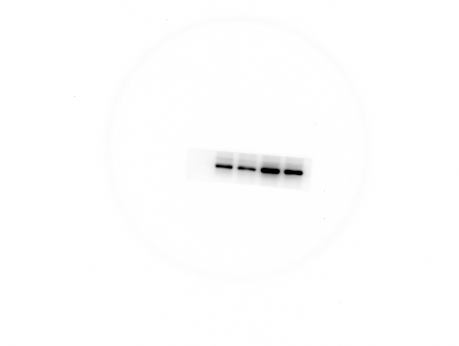
 HEC-1A CDK2 HEC-1B CDK2


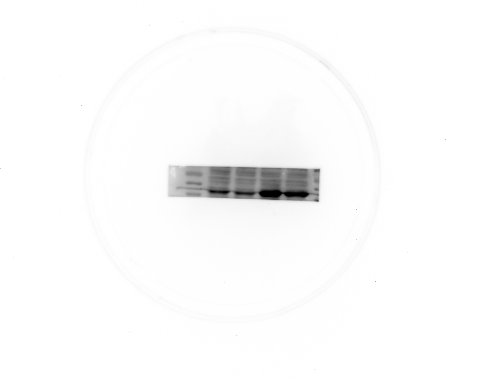

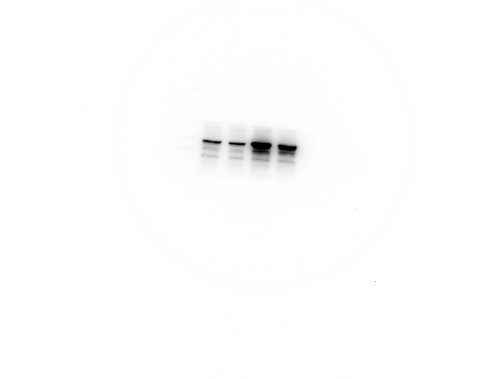
HEC-1A CyclinE1 HEC-1B CyclinE1


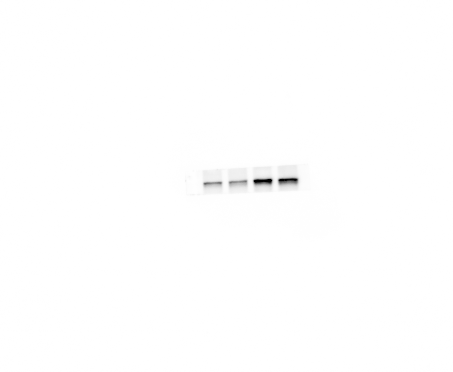

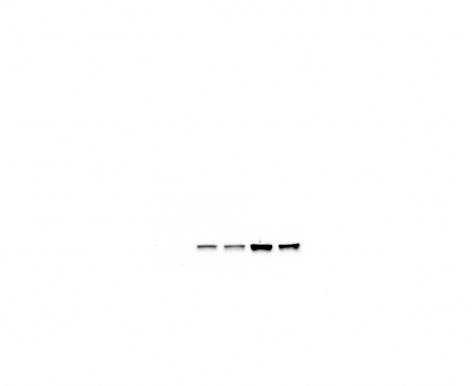
HEC-1A CyclinA HEC-1B CyclinA


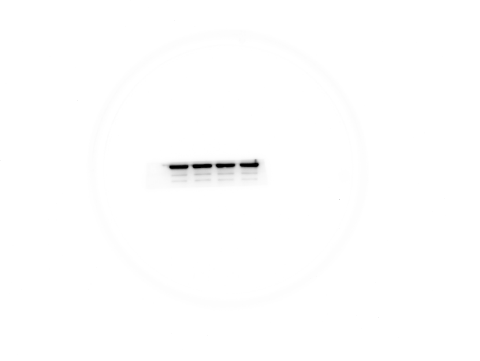

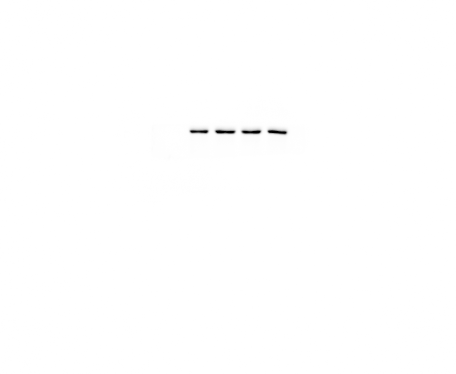
HEC-1A CAPDH HEC-1B GAPDH

Figure 4E


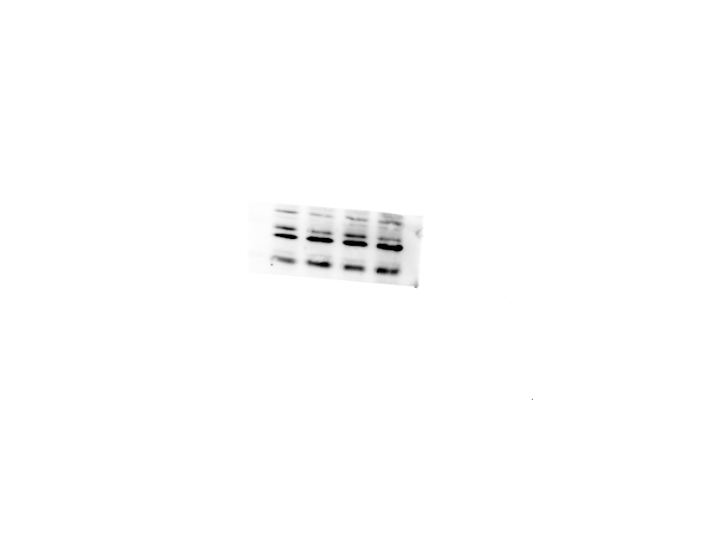

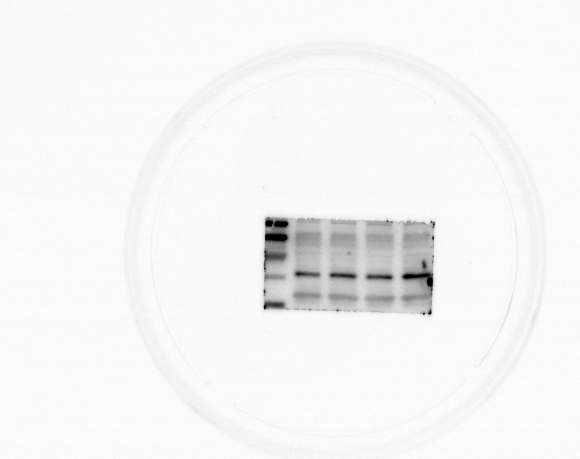
HEC-1A PUMA HEC-1B PUMA


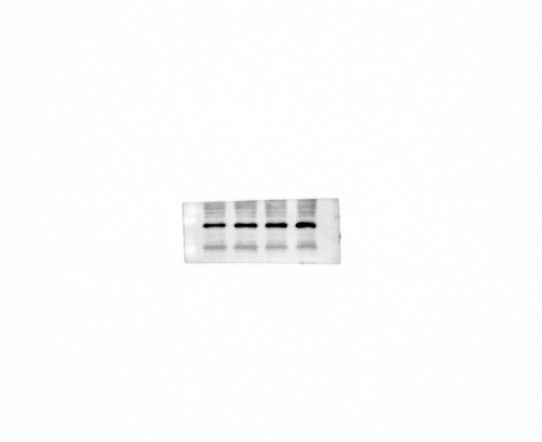

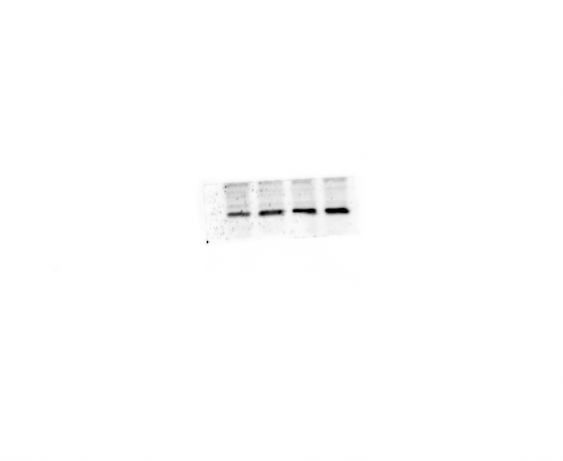
HEC-1A Bax HEC-1B Bax


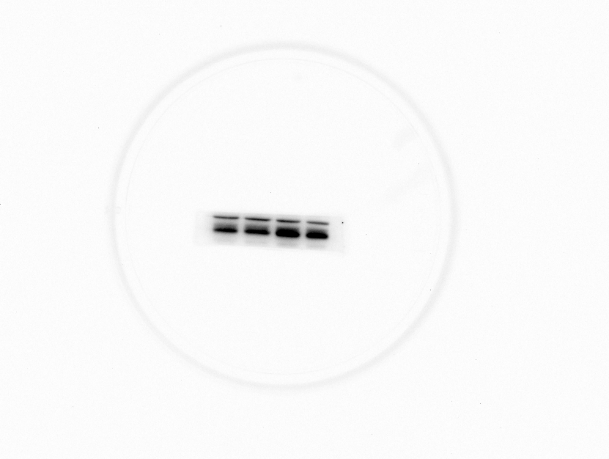

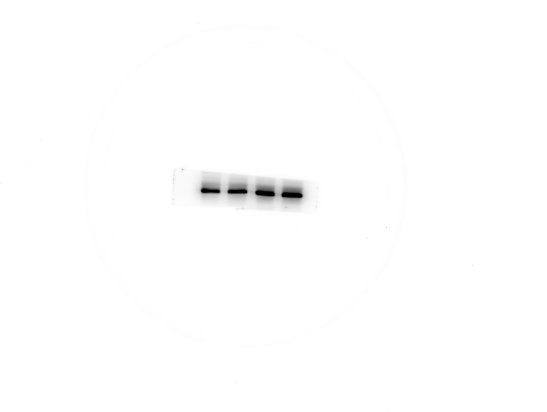
HEC-1A Caspase-3 HEC-1B Caspase-3


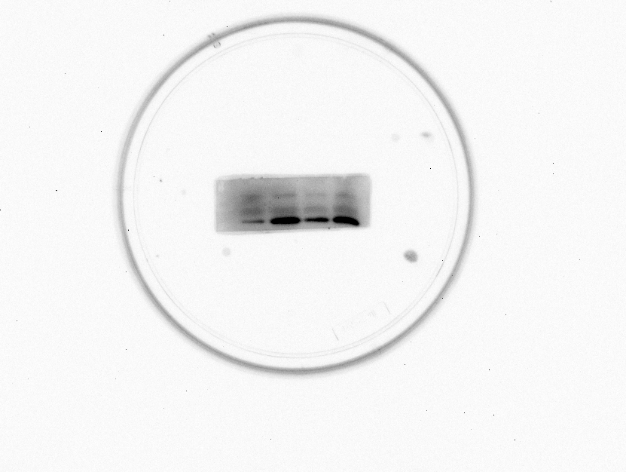

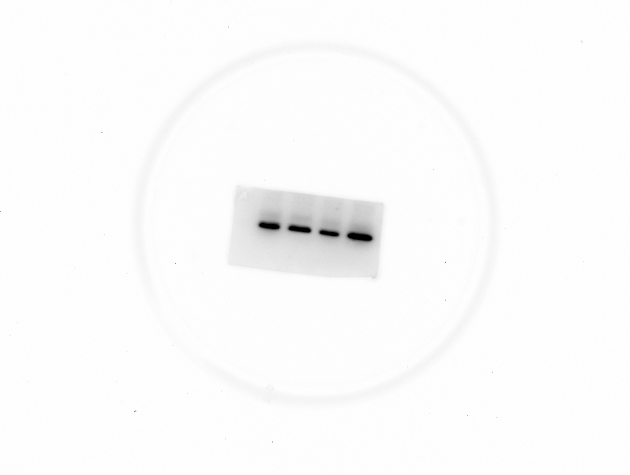
HEC-1A c-Caspase-3 HEC-1B c-Caspase-3


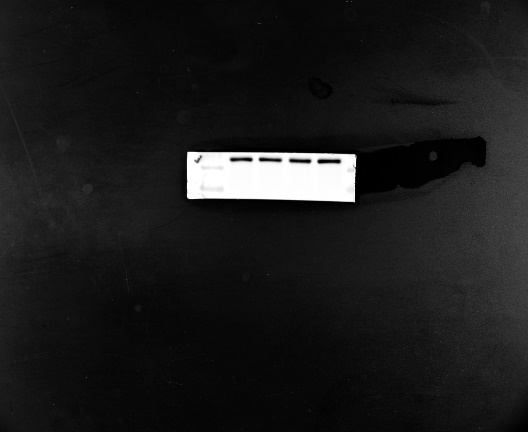

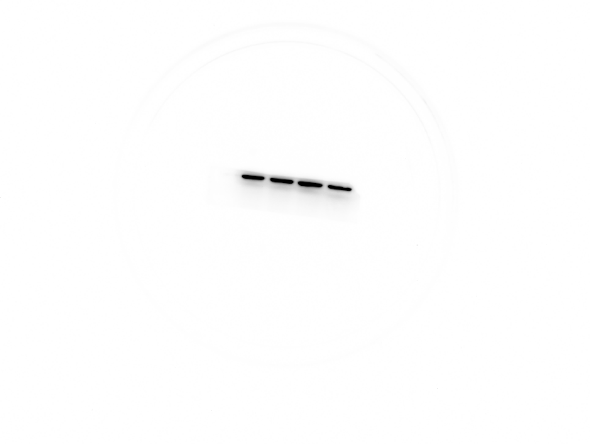
HEC-1A GAPDH HEC-1B GAPDH

Supplementary Figure S2 K




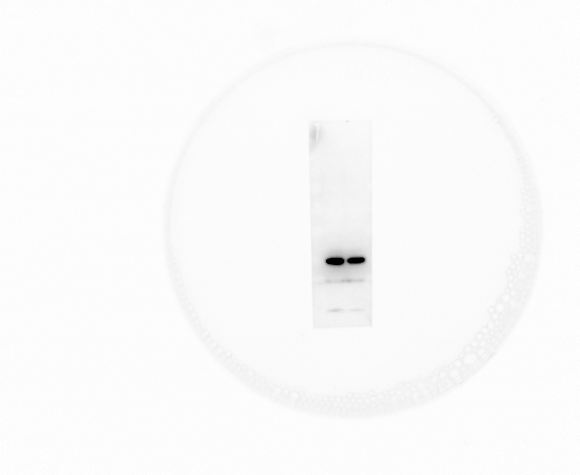

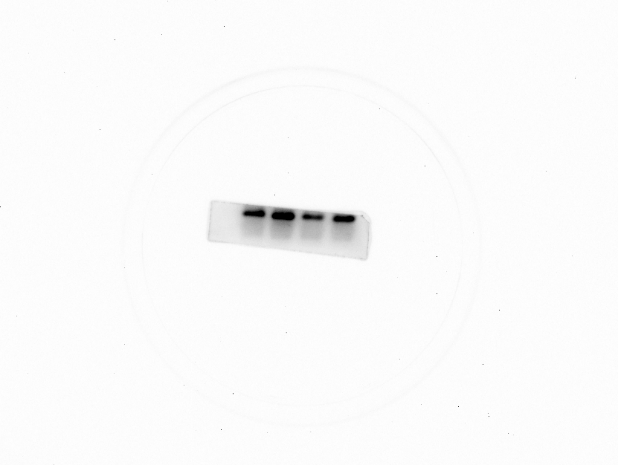
HEC-1B P21 CDK4 CDK6


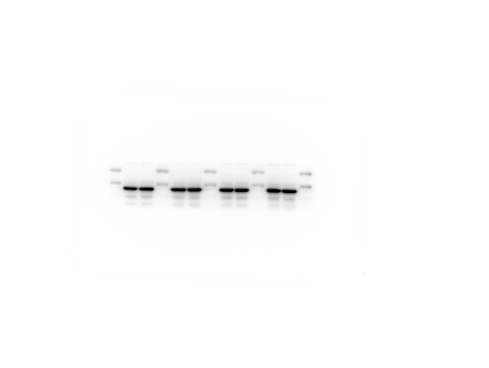



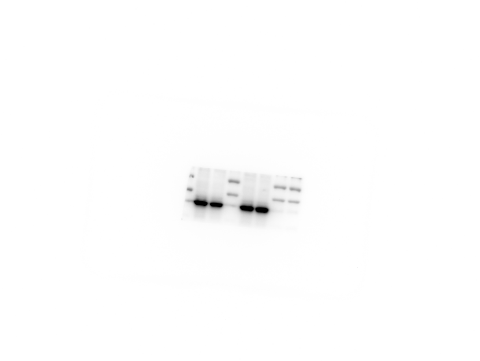
CDK2 CyclinD1 GAPDH


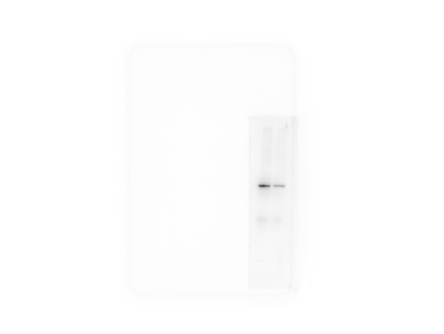

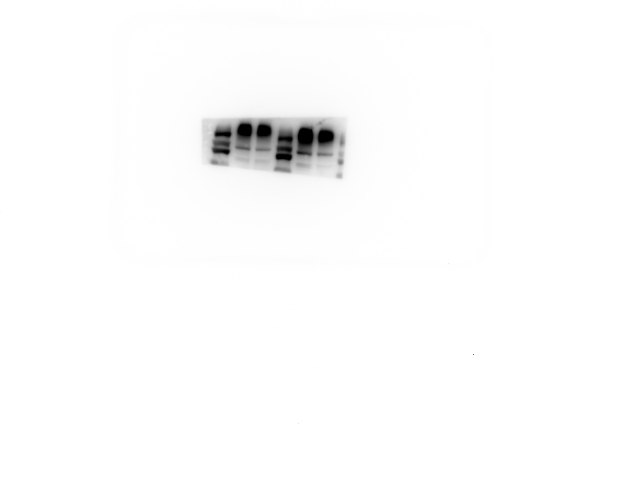

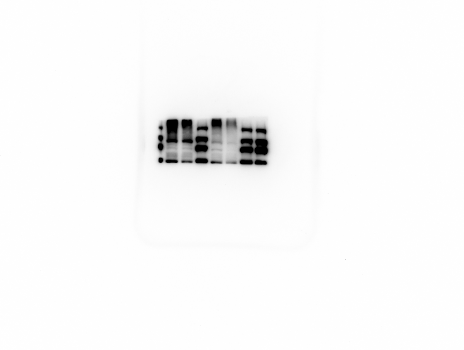
Rb p-Rb E2F1


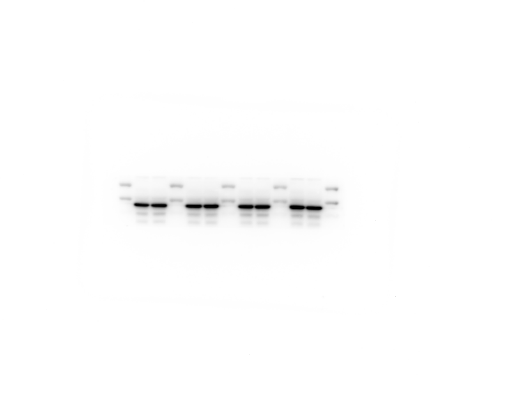

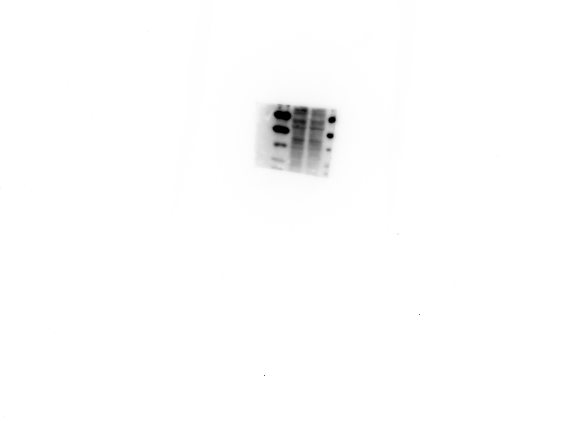

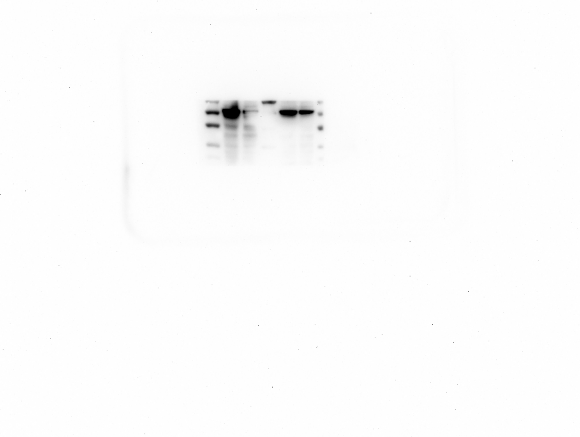
CyclinE1 CyclinA GAPDH

Supplementary Figure S2 M

HEC-1A RAD51 HEC-1B RAD51


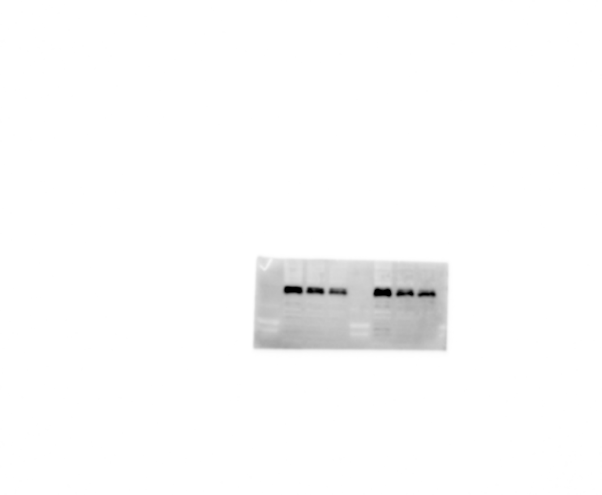


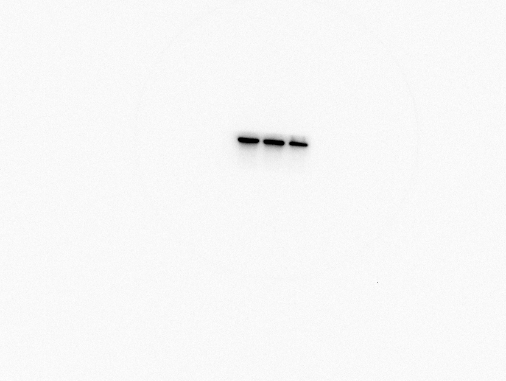

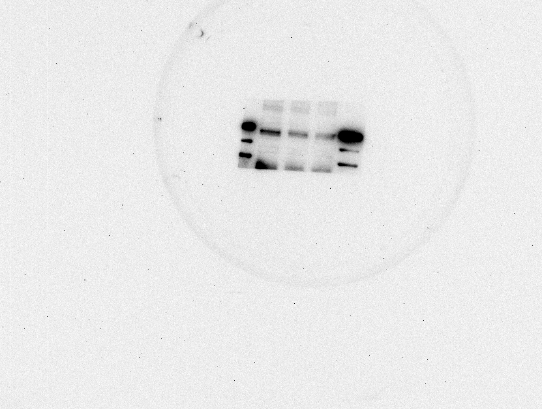
HEC-1A E2F1 GAPDH


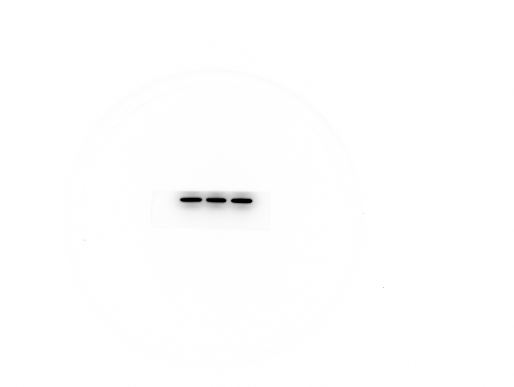

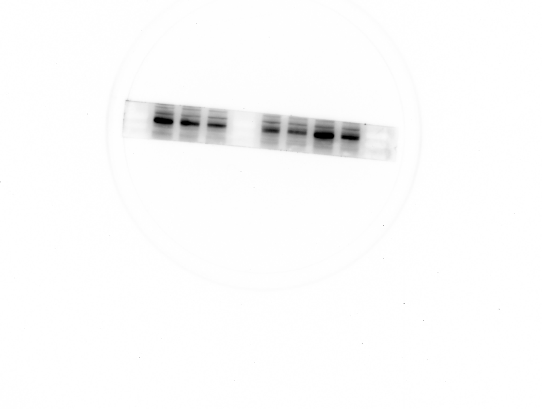
HEC-1B E2F1 GAPDH

Supplementary Figure S2 O

HEC-1A E2F1 HEC-1B E2F1


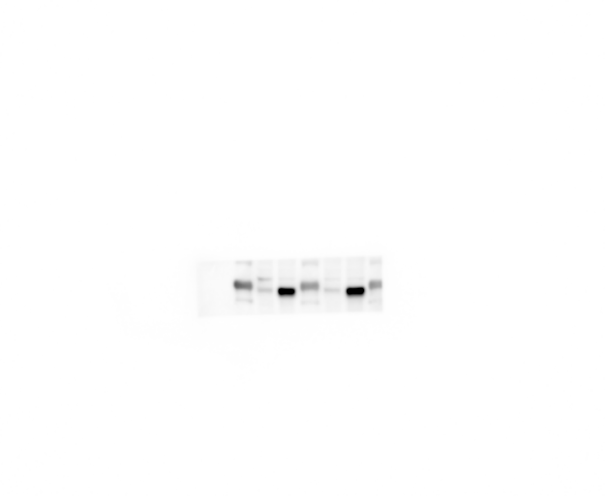


HEC-1A GAPDH HEC-1B GAPDH


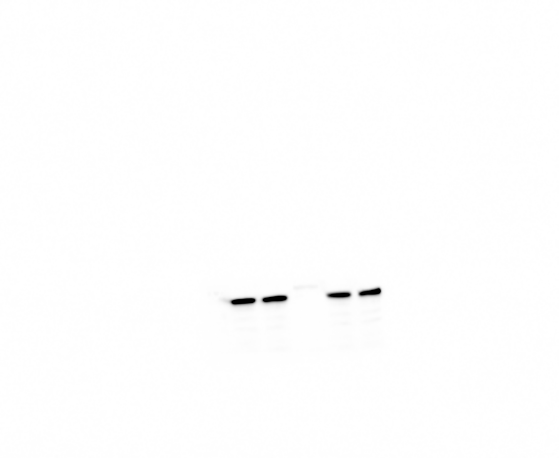


Supplementary Figure S2 Q

HEC-1A RAD51 HEC-1B RAD51


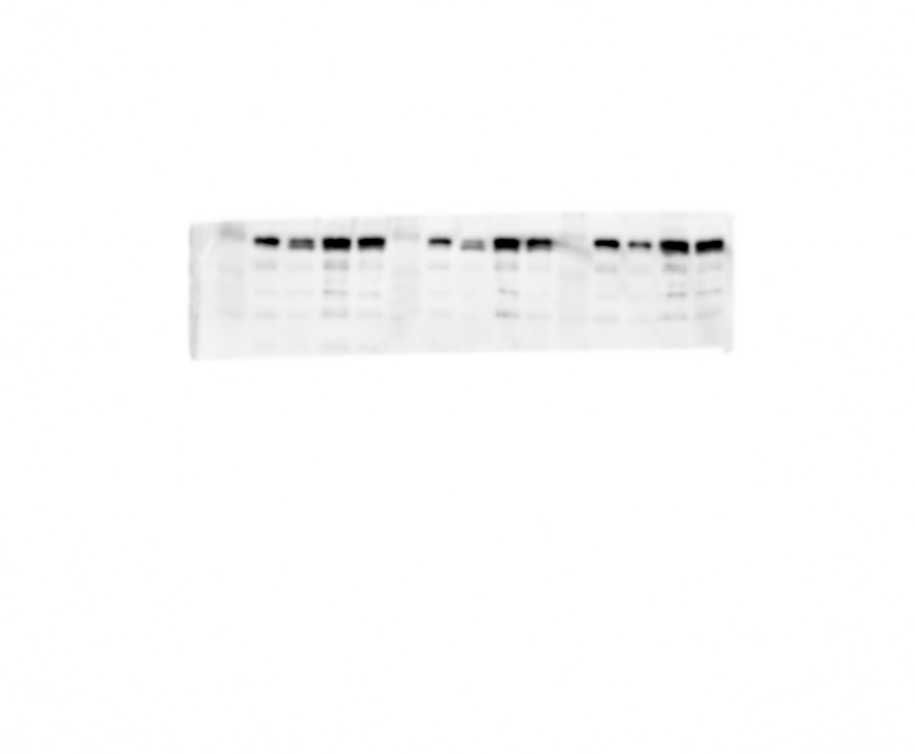


HEC-1A GAPDH HEC-1B GAPDH
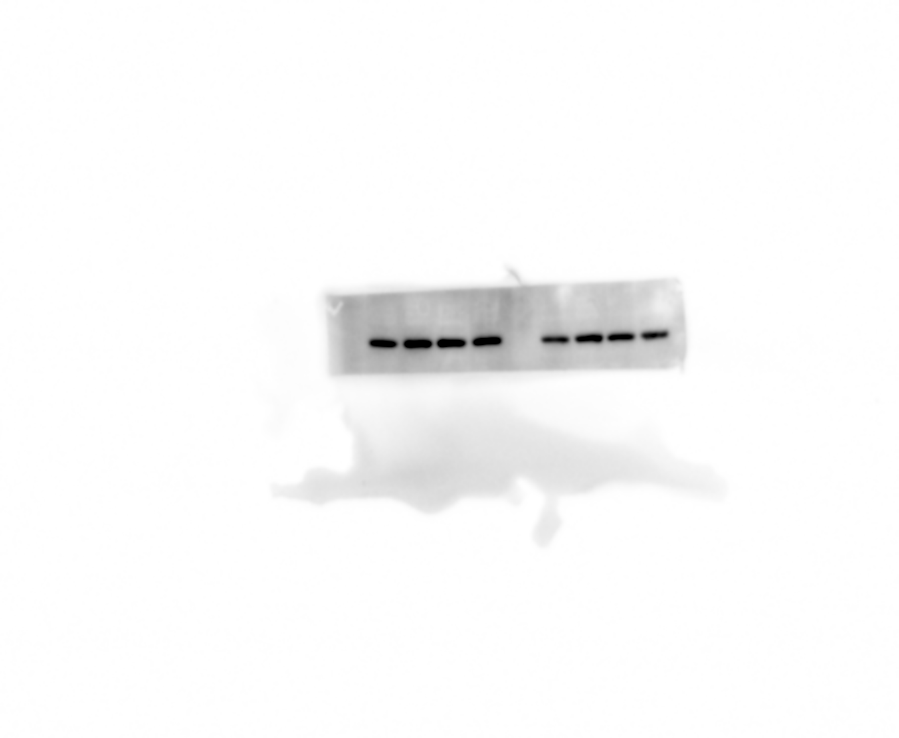


Supplementary Figure S2 G

HEC-1A RAD51 HEC-1B RAD51


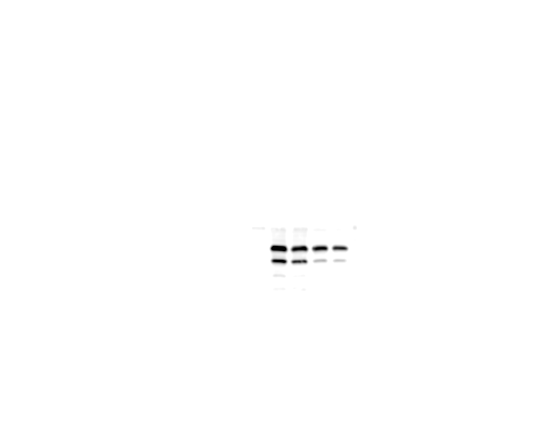

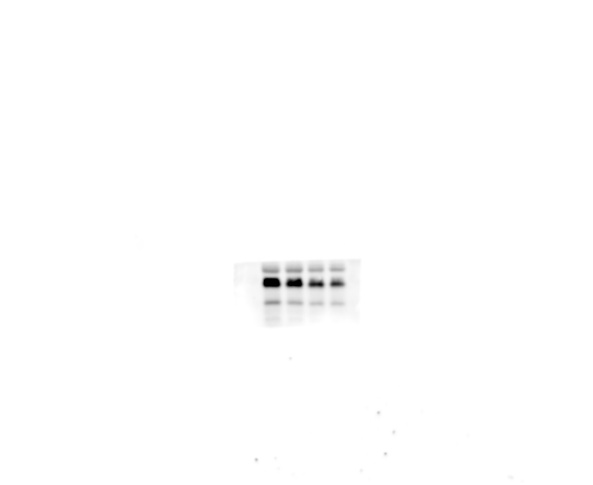


HEC-1A GAPDH HEC-1B GAPDH


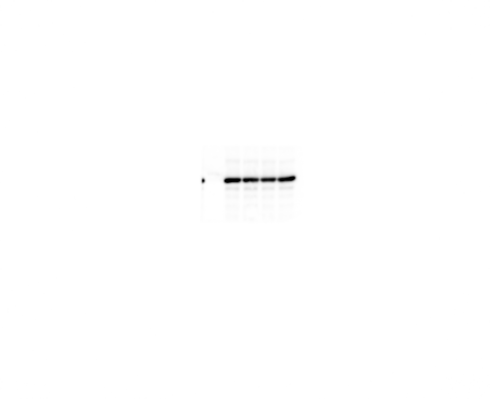

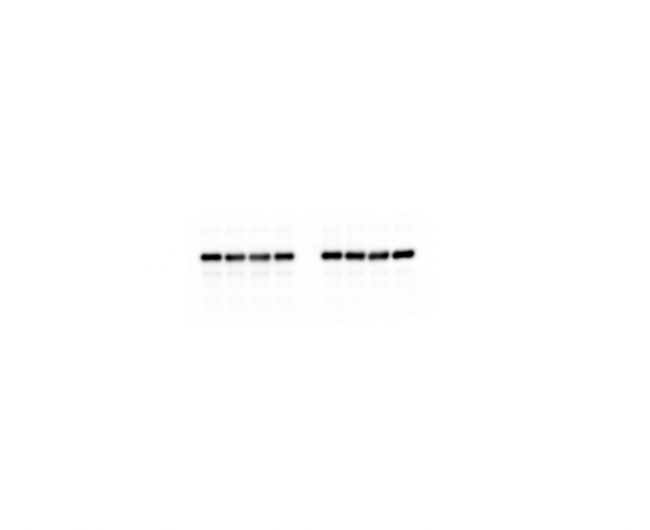


Supplementary Figure S2 H

24h HEC-1A γ-H2AX HEC-1B γ-H2AX


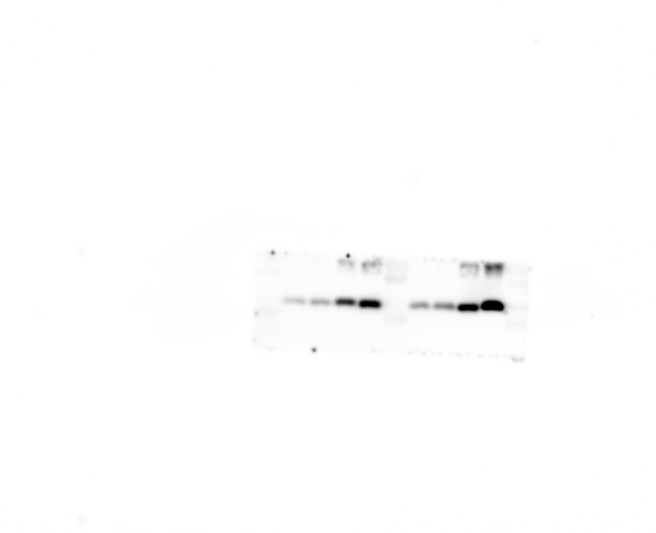

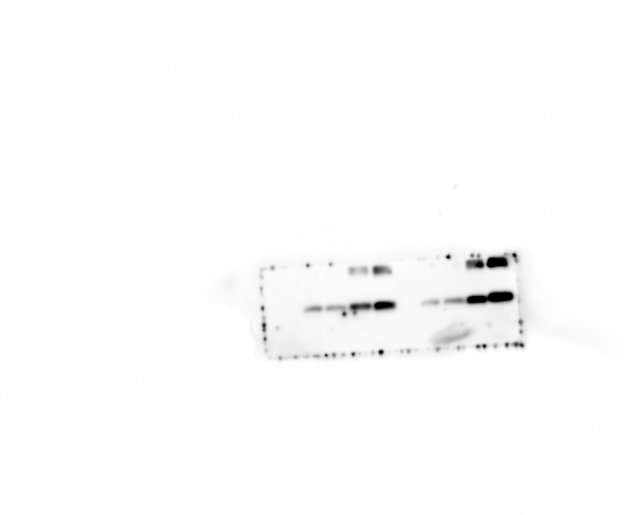


72h HEC-1A γ-H2AX HEC-1B γ-H2AX


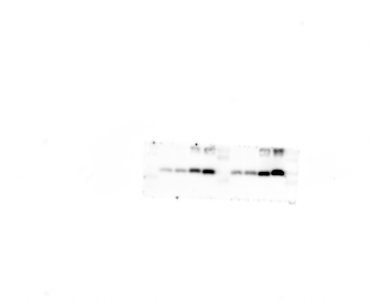

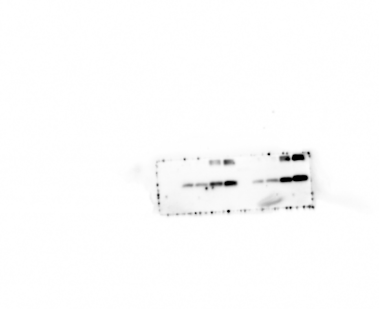


24h HEC-1A GAPDH HEC-1B GAPDH


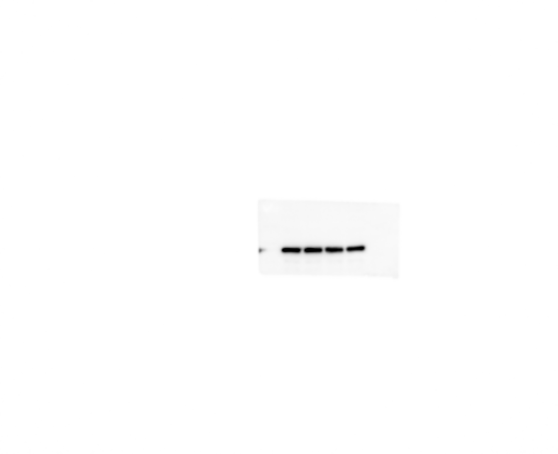


72h HEC-1A GAPDH HEC-1B GAPDH

Supplementary Figure S3 B

HEC-1A p21 HEC-1B p21

HEC-1A GAPDH HEC-1B GAPDH

Supplementary Figure S4 E

HEC-1A Bax

HEC-1B Bax

HEC-1A Caspase-3 HEC-1A c-Caspase-3

HEC-1B Caspase-3 HEC-1B c-Caspase-3

HEC-1A γ-H2AX

HEC-1B γ-H2AX

HEC-1A GAPDH HEC-1B GAPDH

Supplementary Figure S5 F

HEC-1B BRCA1 HDAC1

ATF3 GAPDH

Supplementary Figure S5 I

HEC-1A BRCA1 HEC-1B BRCA1

HEC-1A HDAC1 HEC-1B HDAC1

HEC-1A ATF3 HEC-1B ATF3

HEC-1A GAPDH HEC-1B GAPDH

Supplementary Figure S5 L

HEC-1A HDAC1 HEC-1B HDAC1

HEC-1A GAPDH HEC-1B GAPDH

Supplementary Figure S5 N

HEC-1A BRCA1 HEC-1B BRCA1

HEC-1A GAPDH HEC-1B GAPDH

Supplementary Figure S5 C

HEC-1A BRCA1 HEC-1B BRCA1

HEC-1A GAPDH HEC-1B GAPDH

Supplementary Figure S6 F

HEC-1A Bax HEC-1B Bax

HEC-1A Caspase-3 HEC-1A c-Caspase-3

HEC-1B Caspase-3 HEC-1B c-Caspase-3

HEC-1A γ-H2AX

HEC-1B γ-H2AX

HEC-1A GAPDH HEC-1B GAPDH

Supplementary Figure S7 B

HEC-1A PUMA HEC-1B PUMA

HEC-1A GAPDH HEC-1B GAPDH
